# Smell compounds classification using UMAP to increase knowledge of odors and molecular structures linkages

**DOI:** 10.1371/journal.pone.0252486

**Published:** 2021-05-28

**Authors:** Marylène Rugard, Thomas Jaylet, Olivier Taboureau, Anne Tromelin, Karine Audouze

**Affiliations:** 1 T3S, Inserm UMR S-1124, Université de Paris, Paris, France; 2 Inserm U1133, CNRS UMR 8251, Université de Paris, Paris, France; 3 Centre des Sciences du Goût et de l’Alimentation, AgroSup Dijon, CNRS, INRAE, Université Bourgogne Franche-Comté, Dijon, France; The University of Alabama in Huntsville, UNITED STATES

## Abstract

This study aims to highlight the relationships between the structure of smell compounds and their odors. For this purpose, heterogeneous data sources were screened, and 6038 odorant compounds and their known associated odors (162 odor notes) were compiled, each individual molecule being represented with a set of 1024 structural fingerprint. Several dimensional reduction techniques (PCA, MDS, t-SNE and UMAP) with two clustering methods (k-means and agglomerative hierarchical clustering AHC) were assessed based on the calculated fingerprints. The combination of UMAP with k-means and AHC methods allowed to obtain a good representativeness of odors by clusters, as well as the best visualization of the proximity of odorants on the basis of their molecular structures. The presence or absence of molecular substructures has been calculated on odorant in order to link chemical groups to odors. The results of this analysis bring out some associations for both the odor notes and the chemical structures of the molecules such as “woody” and “spicy” notes with allylic and bicyclic structures, “balsamic” notes with unsaturated rings, both “sulfurous” and “citrus” with aldehydes, alcohols, carboxylic acids, amines and sulfur compounds, and “oily”, “fatty” and “fruity” characterized by esters and with long carbon chains. Overall, the use of UMAP associated to clustering is a promising method to suggest hypotheses on the odorant structure-odor relationships.

## Introduction

Odorant molecules are largely used in food, cosmetic and perfumes [1, 2]. Moreover, the extra-nasally expression of ORs receptors suggest their potential therapeutic interest [3].

The olfactory system can discriminate a large range of odorants of different shapes, sizes, and chemical functions [4]. The discriminatory capacity is carried out through various processes. The olfactory perception begins at the olfactory epithelium level with the activation of olfactory receptors (ORs) by the binding of odorants. The ORs are mainly expressed in olfactory cilia of the sensory olfactory neurons (OSNs); the activation of ORs triggers the transmission of signals by the OSNs to the olfactory bulb before to be distributed to other regions of the brain such as the piriform cortex [5–8].

There are currently about 7000 odorant molecules reported [9], while number of odors able to be perceived is currently unknown, but could reach 1 trillion [10]. Besides, there are less than 2000 of functional ORs in mammals as a whole (about 400 in Human) [11]. Hence, the olfactory perception and discrimination of a such huge number of odors by a limited number of functional ORs is due to involving a combinatorial coding. The combinatorial coding is based on the fact that a single odorant is recognized by several receptors and that a single odorant receptor recognizes several odorants. So, the odor quality of different odorants are encoded by different combinations of receptors [12, 13].

Obtaining a reliable description of the odors by the overall sensory is complicated as emotional context has been reported to be very strongly associated with olfactory information [14, 15]. Indeed, studies of brain activity have shown that exposure to olfactory stimuli activates some brain structures of the limbic system linked to emotions, learning and memory. Hence, odors are difficult to describe verbally, and the words used depend on the context, the familiarities with odor, and culture-specific experiences [16, 17]. Nevertheless, the verbal description of odor remains a main way to characterize the olfactory biological activity of odorants in Human [18, 19]. According to a medicinal chemistry approach of odor perception, matching ligands to ORs are critical for understanding the olfactory system. Indeed, olfactory receptor deorphanization should aid to understand how the molecular properties of odorant molecules act on the receptor activation. However, ligands have been published for nearly 10% of the approximately 400 functional human ORs [20, 21]. Because of the difficulty to deorphanize the ORs by experiment, *in silico* approaches are a promising way, as well by ligand (odorants) approaches as by target (ORs) approaches. Assuming that odorants detected by the same OR have related structures [22], several studies have been carried out to explore relationships between the structure of odorants and their receptors by creating different models using different approaches such as Quantitative Structure–Activity Relationship (QSAR) [23], neural networks [24] and docking [25]. For example, previous studies have developed predictive models based on neural networks for camphoraceous and fruity odors [26], or using artificial intelligence [27], for example by combining fuzzy logic with Kohonen neural networks [28–30]. These hybrid methods have shown their ability to establish robust structure-odor relationships models on different series of molecules, allowing a clustering of the odors for a set of test molecules with a prediction rate of over 70%. The study of several ORs were also performed through mutagenesis, molecular modelling, and functional expression and led to identify the structure of binding site, improving the knowledge of structure-functions relationships of the ORs [25, 31–33]. Other strategies were to develop integrative systems biology based-models using existing knowledge such as ligand-protein associations and protein-protein interactions in order to decipher the human odorome [34]. Nevertheless, despite significant advances, establishing the link between odors and molecular structures remains largely unresolved and challenging [35–37]. Our study focuses on the relationship between the structures of a large set of smell compounds and their odors. For this purpose, we built a dataset comprising more than 6000 smell compounds associated with their smell description by compiling information available in several databases [38, 39]. The structural information of the molecules was encoded into fingerprints, and a computational study aiming to analyze and visualize the smell compounds distribution in their chemical space was performed. Four-dimensional reduction techniques combined with two clustering methods were tested in order to select the most suitable approach for the present dataset. Two classical dimensional reduction methods, Principal Component Analysis (PCA) and Multidimensional Scaling (MDS), and two more recent approaches, the t-Distributed Stochastic Neighbor Embedding (t-SNE) [40], and the Uniform Manifold Approximation and Projection (UMAP) [41] were chosen. After data reduction, clustering analyses were performed individually either by k-means or by agglomerative hierarchical clustering (AHC) using the 2-dimensional space coordinates defined by each dimension reduction techniques. Then, an analysis of the distribution of odor notes and chemical functions / molecular substructures represented in the different clusters was performed. The association of the UMAP method with clustering appeared to be a relevant combination to discriminate the relationships between the structures of molecules and their odors.

## Materials and methods

### Data of smell compounds, odor notes and ORs

For this study, a dataset of 6038 smell compounds and 162 odor notes (of which “odorless”) having at least 5 occurrences [42] was extracted and compiled from the databases "The Good Scents Company" (access 23/01/19) [39] and "Flavor Base" (9^th^ Edition) [38]. Data can be available upon request.

### Encoding molecular structures into fingerprints

Each molecular structure was encoded into Extended-connectivity fingerprints (ECFP), i.e. in binary vector: the presence of a given function/substructure in the compound is represented by 1, while its absence is represented by 0 [43, 44]. In these fingerprints, substructures are generated by considering each atom and their neighborhood on several circular layers (up to a given diameter/radius). To calculate them, KNIME software (v 3.6.2) was used with the following parameters: radius = 2, allowing to obtain fingerprints equivalent to Extended-connectivity fingerprints 4 (ECFP4). ECFPs are fingerprints specially developed to seizure molecular features necessary to molecular activity and particularly suited to Tanimoto similarity methods [45]. More specifically, ECFP4 are known for their efficiency [45, 46], and are among the best on small molecules benchmarks [47]. The use of bits number = 1024 associated to these fingerprints, makes it possible to obtain a fairly precise molecular structure for the study [48].

In addition, sixty-two molecular substructures of the smell compounds were computed with KNIME in the aim to identify potential relations between the odor notes and the chemical functional groups of molecules.

### Dimension reduction from the 1024-bit fingerprints

To visualize the encoded smell compounds, four-dimensional reduction techniques were applied: PCA, MDS, t-SNE and UMAP. The PCA, MDS and t-SNE methods were performed using the R software (v 4.0.2) using several packages such as FactoMineR, stats and labdsv, while the UMAP method (v 0.4) [49] was applied using Python 3.7.6 with the package ’umap-learn’. The PCA is a multivariate analysis method, that allows to extract the most important information by an orthogonal transformation to generate correlated variables with new linearly independent variables called principal components [50]. MDS is a network localization technique that maps the similarity or the dissimilarity of pairs of objects from a dataset. The similarity/dissimilarity is converted into distances between points in a two-dimensional space [51, 52]. The t-SNE method is an improved version of the Stochastic Neighbor Embedding (SNE). Like the SNE, the t-SNE measures the similarity between pairs of objects of the high dimensional data and of the two-dimensional embedding. Therefore the t-SNE generates a two-dimensional embedding using gradient descent to minimize the Kullback-Liebler divergence between the vector of similarities between pairs of objects in the high dimensional data and the similarities between pairs of objects in the embedding [53]. The UMAP method is a recently developed dimension reduction technique [41] that allows to precisely capture the non-linear structure of large data sets. UMAP is a manifold technique constructed from a theoretical framework based on Riemannian geometry and algebraic topology. The manifold theory considered the following key concept: a manifold is a space where points are gathered according to their Euclidean distances, so forming a continuous map. According the proximities between the points, differentiable manifolds can be identified on the map [54]. Then, UMAP uses local manifold approximations and patches together their local fuzzy simplicial set representations to construct a topological representation of the high dimensional data. Given some low dimensional representation of the data, an equivalent topological representation can be built using a similar process. Then, the layout of the data representation is optimized in the low dimensional space allowing minimization of the cross-entropy between the two topological representations [49]. Globally, UMAP starts by calculating the distances between each point. It then considers, for each point, its n closest neighbors and assigns a weight (probability) of link between the point considered and its n neighbors. From this, UMAP builds a weighted graph and then uses a “force-based layout” algorithm on it to project and represent data optimally at low dimensions. The UMAP advantage is that it is customizable in order to be adapted to its own data. For example, several parameters can be modified after data integration: the distance calculation method, the number of the neighbors, the minimum distance for grouping points at low dimensions, or the desired number of dimensions. To calculate distances/similarities between fingerprints, three metrics seem to be the most suitable: the Tanimoto/Jaccard index, the Dice index and the Cosine coefficient [55]. The Jaccard/Tanimoto index, which represents the fraction of bits shared between 2 fingerprints, gave the best results in preliminary assays on our data, and was therefore selected for our study.

The objective of our visualization was to obtain a compromise between local and global information, in order to suitably perceive the emergence of groups containing structurally close molecules. For that, the appropriate choice of the values of the number of neighbors and the minimum distance is crucial. The “number of neighbors” parameter allows to balance local versus global structure in the data. So, at low values, UMAP concentrates on very local structure even to the detriment of the global picture. But at higher values, UMAP focuses on larger neighborhoods of each point but loses detail structure. The “minimum distance” parameter controls the distance with which the points are grouped together in the low dimensional representation. At low values, there are clumpier embeddings between the points and at higher values, the points are much less grouped and UMAP preserves more the broad topological structure. After testing of different values of these two parameters, the number of neighbors and the minimum distance were fixed to 15 and 0, respectively.

### Visualization, clustering and structure-odor analysis

AHC and k-means clustering were carried out from the reduced dimensions obtained with the four previous techniques, to group structurally similar molecules. These clustering were done using R (v 4.0.2) on the two dimensional data to avoid problems associated with high-dimensional clustering [56]. For AHC, Euclidean distance matrix 2 to 2 of each molecule was calculated with the aggregative criterion "ward.D2", which seeks to minimize the intra-class inertia and maximize the inter-class inertia. The two closest classes were thus successively grouped until obtaining a complete clustering tree. Hierarchical clustering is simple and easy to use whatever the form of similarity or distance [57, 58]. This technique has great flexibility with regard to a level of granularity, and is applicable to any attribute types [58]. However, the merging of clusters is definitive. Therefore it is not possible to correct erroneous decisions [57]. For the k-means clustering, several numbers of centroids corresponding to the numbers of clusters were tested and the points of the dataset were assigned to its nearest cluster at each iteration. All points of the same cluster were averaged and new centroids were recalculated. Cluster centroids were improved at each iteration until there is no more changes [59]. The k-means algorithm is known to be sensitive to outliers, and less efficient with clusters that are not hyper-spheres [57]. To choose the optimal number of clusters, the intra-cluster variability was analyzed. The aim was to have a low intra-cluster variability to obtain homogeneous groups, but high enough so that the population within each cluster is sufficient.

**Fig 1:**
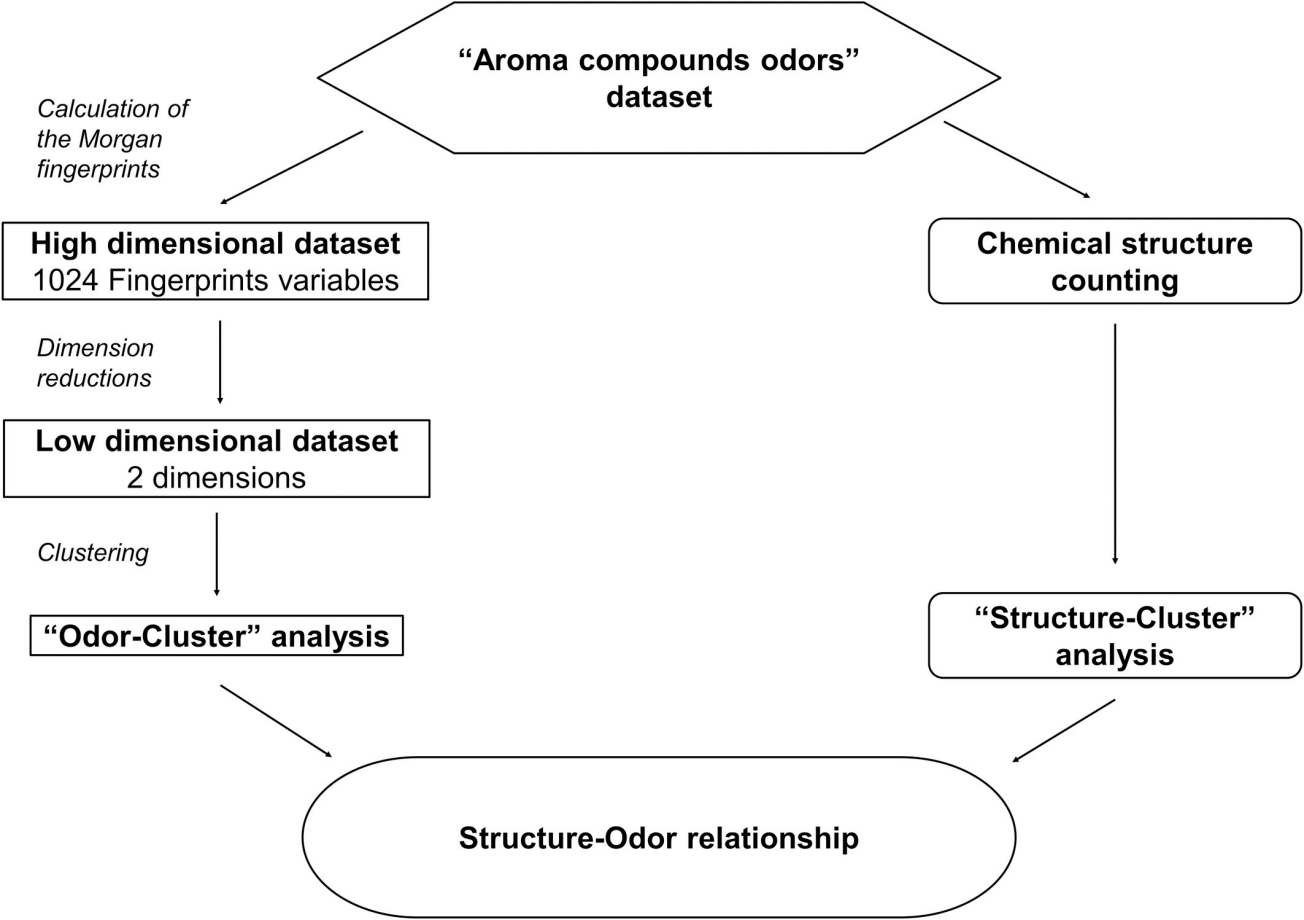
Representation of the workflow. On the left, reduction of the high dimensional space defined by the fingerprints and clustering; on the right, molecular substructures calculation.

Once the clusters were defined, either by AHC or k-means, we first looked into the distribution of odor notes across the clusters. Then the chemical groups/functions of the molecules belonging to the different clusters were investigated. The overall setting up protocol is described in Fig 1.

## Results

### Overview analysis of the dataset of odorant compounds

**Fig 2:**
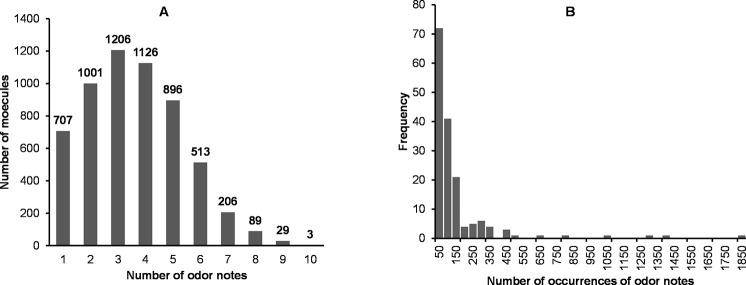
Distribution of the odor notes and the number of their occurrences. A: Histogram of the number of odorants according to the number of odor notes. B: Histogram of the workforce according to the number of occurrences of the odorants.

The dataset encompasses 6038 smell compounds, of which mainly odorants, but also various smell compounds, whose sapid compounds or additives (inorganic salts, amino-acids, peptides, polymers…). Such molecules have little or no volatility, and consequently are unable to reach in vapor phase to the nasal cavity to activate the ORs. Therefore, these compounds are described “odorless”. We identified 261 compounds with these characteristics. Excluding the “odorless” compounds, most odorants are described by 2 to 5 odor notes (Fig 2A). The number of occurrences of the odor notes ranges from 1828 (“fruity”) to 5 (“bland” and “tallow”). Most of the odor notes have less than 150 occurrences (Fig 2B), while only 4 odor notes exceed 1000 occurrences (1828 for “fruity”, 1389 for “green”, 1283 for “sweet”, 1010 for “floral”).

### Dimensions reduction, clustering and visualization of the data

**Fig 3:**
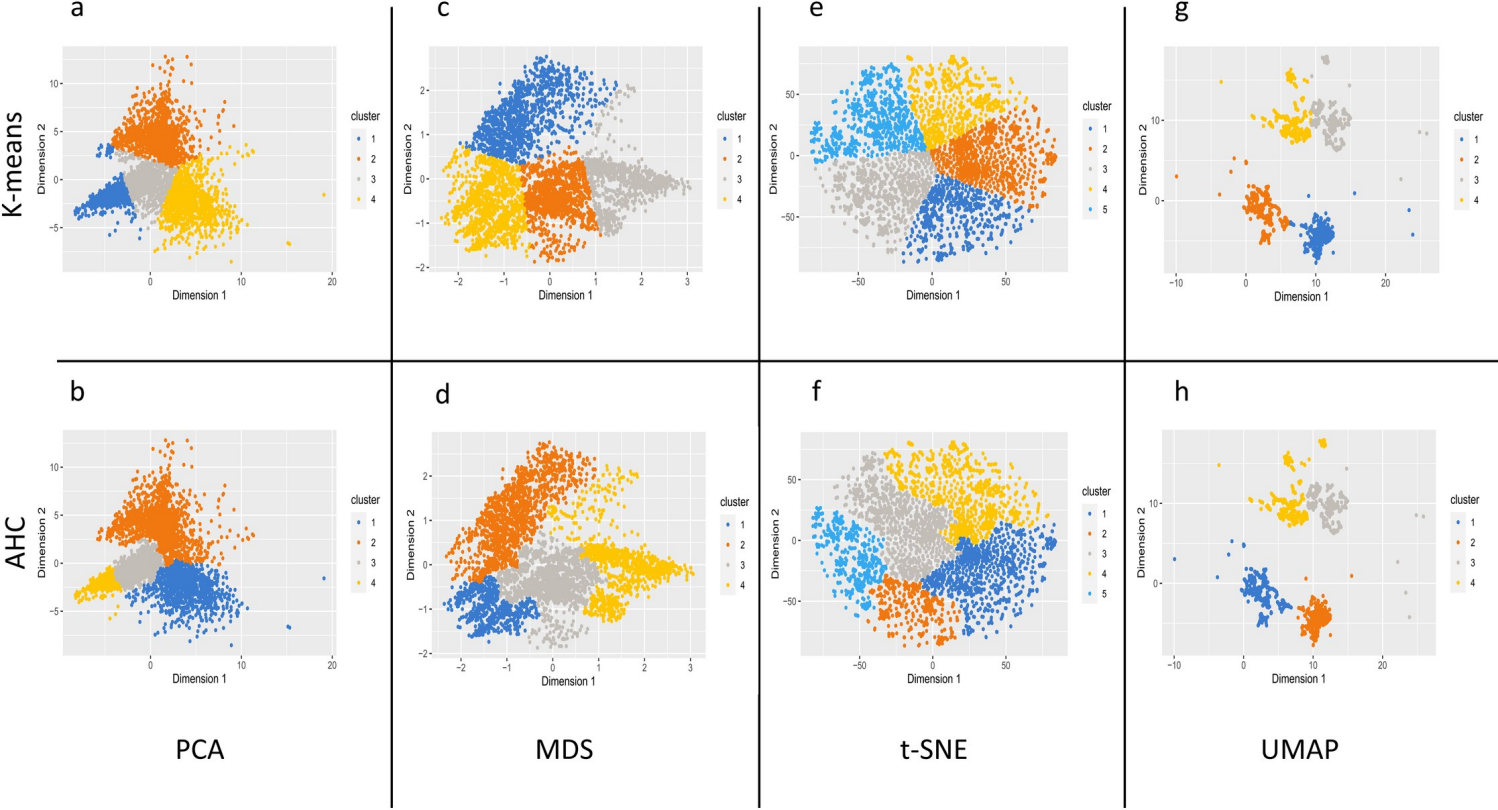
Visualization of the compounds-odors dataset in the 2-two dimensional spaces obtained after dimension reduction using PCA, MDS, t-SNE and UMAP. The data are colored according to the clusters produced by the k-means clustering and AHC that were carried out on the basis of the coordinate in the 2D spaces. The colors allow only to visualize the clusters easily and are specific to each method; there is no correspondence between the colors according to the several methods. The data are reported in S1 Table. (a) Clusters obtained by the PCA k-means approach: the clusters C1a, C2a, C3a and C4a encompass respectively 1523, 1466, 1622 and 1427 smell compounds; (b) Clusters obtained by PCA AHC approach: the clusters C1b, C2b, C3b and C4b encompass respectively 1461, 1756, 1997 and 824 smell compounds; (c) Clusters obtained by MDS k-means approach: the clusters C1c, C2c, C3c and C4c encompass respectively 1312, 1774, 1468 and 1484 smell compounds; (d) Clusters obtained by MDS AHC approach: the clusters C1d, C2d, C3d and C4d encompass respectively 854, 1551, 1970 and 1663 smell compounds; (e) Clusters obtained by t-SNE k-means approach: the clusters C1e, C2e, C3e, C4e and C5e encompass respectively 1008, 1375, 1225, 1122 and 1308 smell compounds; (f) Clusters obtained by t-SNE AHC approach: the clusters C1f, C2f, C3f, C4f and C5f encompass respectively 1480, 636, 1633, 1524 and 765 smell compounds; (g) Clusters obtained by UMAP k-means approach: the clusters C1g, C2g, C3g and C4g encompass respectively 1597, 1344, 1454 and 1643 smell compounds; (h) Clusters obtained by UMAP AHC approach: the clusters C1h, C2h, C3h and C4h encompass respectively 1640, 1584, 1332 and 1482 smell compounds. In each chart, C1, C2, C3, C4 and C5 clusters are depicted respectively in blue, orange, grey, yellow and light blue.

The high-dimensional data provided by the 1024 calculated fingerprints, used to encode the molecular structures of the smell compounds, were reduced to two-dimensional data using four dimensional reduction techniques. Then, the two clustering methods were applied to these 2D space coordinates to group the most similar molecules according to their structure. To determine the optimal number of clusters, an “elbow” curve representing the intra-cluster variability as a function of the number of clusters was done (S1 Fig) for each dimensional reduction technique. As the elbow curve showed a variable optimal number of clusters, a Kelley penalty score was used in addition to precisely determine the optimal number of clusters (S2 Fig). The minimum score is attributed to the optimal number of clusters, which was five clusters for the t-SNE and four clusters for the three other techniques. For our study, the clustering calculations were carried out, following these numbers of clusters, to have a good balance between variability and number of individuals per group. The assignment of smell compounds in the 2-two-dimensional space defined by the calculation of all techniques is shown in Fig 3.

The projection of the PCA, MDS and t-SNE maps did not shown a clear separation. Instead, the UMAP technique revealed a good separation of the four groups. The color representation of the compounds by clusters displayed well defined areas using the four-dimensional reduction techniques. Nevertheless, the areas defined by each of the clustering methods were not identical when applied to a same dimensional reduction approach. Indeed, each of the two clustering methods could separate differently the 2D-spaces. Thus, to assess the homogeneity of clustering between the 2 clustering methods, intersection of two clusters were computed using the following equation:

Cx(Mk‐means)∩Cy(MAHC)

where x and y were cluster numbers, M referred to the dimensional reduction methods, and ∩ was the mathematical intersection operator. In other words, it measured the number of molecules that belonged to two clusters obtained with the two clustering methods. For example, C1a(PCA k-means)∩C1b(PCA AHC) encompassed 16 common molecules. In addition, the dendrograms from each of the four AHC studies allowed to determine which clusters were aggregated, and thus which clusters were closer (S3 Fig). With PCA-AHC, clusters 1 and 2, and clusters 3 and 4 aggregated. About the MDS-AHC, clusters 4 and 2, and clusters 1 and 3 merged. The t-SNE-AHC technique showed that clusters 3 and 5 aggregated together, and then with cluster 4 in one side whereas clusters 1 and 2 joined in the other side. Finally, on the UMAP-AHC, clusters 1 and 2 were aggregated, as well as clusters 3 and 4.

### Analysis of the cluster constituents: structure-odor relationships

#### Odor notes

We performed the analysis of the cluster composition considering two viewpoints: the frequencies of the odor notes carried by the smell compounds, and the number of molecules carrying specific odor notes. More precisely, because the number of occurrences of the odors varied in a large range from 5 to 1828, the direct comparison of the number of occurrences would not be reliable for the less frequent odor notes. Therefore, we considered two ratios (S2 Table):

$$\%\;odor\;notes=\%ON=\frac{number\;of\;occurrences\;of\;an\;odor\;note\;in\;the\;cluster}{total\;number\;of\;occurrences\;of\;this\;odor}$$

$$\%\;odorant\;molecules=\%OM=\frac{number\;of\;occurrences\;of\;an\;odor\;in\;the\;cluster}{number\;of\;elements(molecules)in\;this\;cluster}$$

For example, with the PCA-kmeans approach, there were 1523 molecules in the cluster C1. The most frequent odor note “fruity” had 1828 occurrences in the dataset and 691 in C1:

%ON"fruity"=6911828=37.8%

%OM"fruity"=6911523=45.4%

Besides “beefy” had 20 occurrences in the dataset, and 3 in C1:

%ON"beefy"=320=15.0%

%OM"beefy"=31523=0.2%

Thus, about 38% of “fruity” molecules were gathered in C1 and constituted 45% of this cluster. Part of the “beefy” molecules (3 odorants) were in C1 representing only 0.2% of this cluster. All the frequency values were reported in S2 Table.

**Fig 4:**
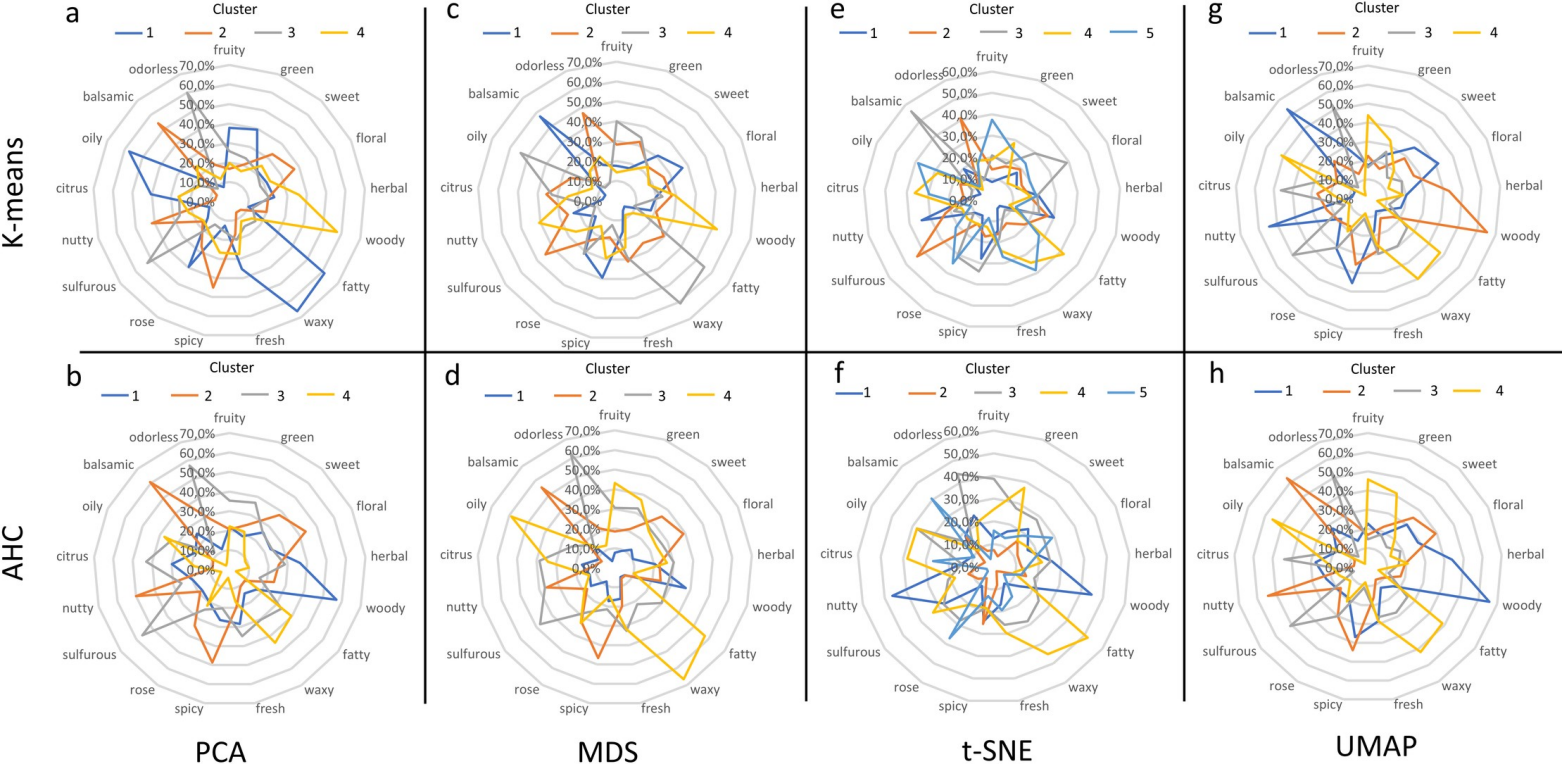
Radar charts of the distribution of the %ON values obtained for the 17 most frequent odor notes across the clusters. (a) Clusters obtained by PCA k-means method; (b) Clusters obtained by PCA-AHC method; (c) Clusters obtained by MDS k-means method; (d) Clusters obtained by MDS-AHC method; (e) Clusters obtained by t-SNE k-means method; (f) Clusters obtained by t-SNE-AHC method; (g) Clusters obtained by UMAP k-means method; (h) Clusters obtained by UMAP-AHC method. In each chart, C1, C2, C3, C4 and C5 clusters are depicted respectively in blue, in orange, in grey, in yellow, in light blue.

To compare the effectiveness of the used techniques to discriminate the odors, radar charts were performed (Fig 4), based on the distribution of the 17 most frequent odor notes across the clusters.

**Fig 5:**
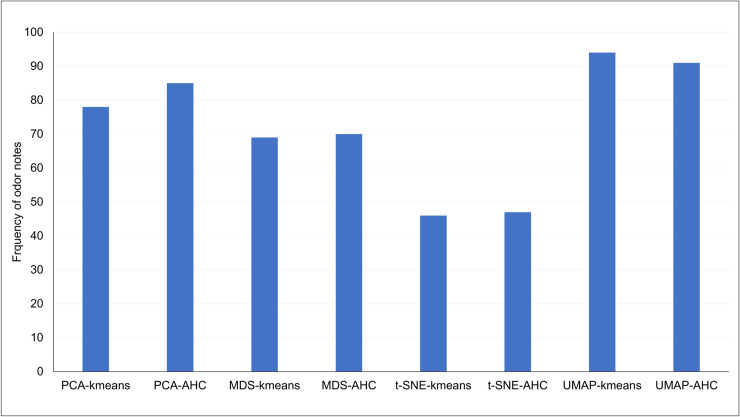
Histogram of the number of odor notes whose %ON is greater than 50 for each technique.

These charts revealed the specificity of several odor notes according to the obtained clusters for each of the dimensional reduction methods. An overview of the specificity of odor notes was summarized by the calculation of the number of odor notes for which %ON is higher than 50. The result is displayed in Fig 5, and showed the greatest discriminant capacity of UMAP whatever the clustering method.

The analysis of the %ON values obtained for the 17 most frequent odor notes provided interesting findings. For clarity, we focused our results on UMAP, the results from the others methods being described in supplementary (S2 Table).

The clusters C1g(UMAP k-means) and C2h(UMAP AHC) were constituted of more than 60% of “balsamic” odor note, as well as “floral”, “spicy”, nutty” and “sweet” notes. Similar profiles were also observed for C2g and C1h (“woody” and “spicy” notes), C3g and C3h (“odorless”, “sulfurous”, “citrus”), and C4g and C4h (“fatty”, “waxy”, “fruity”, “green”). By combining the clusters C1g and C2h, C2g and C1h, C3g and C3h, C4g and C4h and called respectively C1g2h, C2g1h, C3hg and C4hg, the odor notes “woody” and to a lesser degree “spicy” were typical for the molecules belonging to cluster C2g1h (S4B Fig). About 66% of the occurrence of the “woody” note was gathered in cluster C2g1h while “woody” molecules represented about 26% of this cluster. About C2g1h, although it contained about 30 different odors notes, more than 90% of the molecules carried the odor notes “sandalwood” and “cedar” (S2 Table). Additionally, 54 molecules of C2g1h carried both the odor notes “woody” and “spicy” constituted near to 10% of this cluster. “Spicy” note was more frequent in the cluster C1g2h (representing 12% of this cluster). However, “balsamic” was the odor the most represented in C1g2h (66%, S4A Fig). Besides, “nutty” (%ON 54%), “floral” (%ON 39%) and “sweet” (%ON 35%) notes were specifically more frequent in C1g2h comparing to the three other clusters. The cluster C3hg (S4C Fig) put together “sulfurous” and “citrus” odor notes (%ON 51 and 44% respectively). In addition, more than 60% of the occurrences of “mustard”, “garlic”, “onion” and “alliaceous” were in this cluster, whereas “bergamot”, “lemon”, “orange”, “mandarin” were also well represented (S2 Table). Additionally, there was about 100 odorless compounds in C3hg. Finally, the odor notes “oily”, “waxy”, “fatty”, “fruity” and “green” bring together the main part of their occurrences in cluster C4gh (S4D Fig). The “fruity” molecules represented 57% of the cluster C4gh while “fruity” was often associated to another odor note in the odor description, especially to “green” (21%), and also to “apple” (11%). There were also some “fruity-fatty” and “fruity-waxy” associations (5 to 8%).

As presented above, several molecules could belong to intersections between two clusters, noted Cx(UMAP k-means)∩Cy(UMAP AHC). Several of these overlapping clusters corresponded to similar areas of the 2D-spaces, and the belonging molecules were sharing the same odor notes. At the difference, some clusters parts were placed far from the main area of the other elements related to the same cluster. The composition of clusters calculated on the basis of UMAP coordinates were particularly well maintained across k-means and AHC clustering methods. Only 238 molecules were switched to another cluster. The areas C1g ∩ C1h, C1g ∩ C3h, C3g ∩ C4h, C4g ∩ C3h included respectively 44, 15, 158 and 21 molecules. C3g gathered more than “sulfurous” and “odorless” molecules, while the molecules belonging to C4h were characterized by “fruity”, green”, “waxy” and “fatty” notes. The group C3g∩C4h contained nor “sulfurous” nor “odorless” molecule. In opposite, “green” molecules constituted almost three quarters of this group, while “fruity” was shared by more than one third of the molecules. C1g ∩ C1h shared more than one third of molecules with the odor “floral” and the odor “sweet”. For the C3g ∩ C4h area, a large majority of molecules carried the odor “green” (123 molecules). And the area C4g ∩ C3h encompassed 11 molecules with the fruity odor. It was therefore the molecules carrying the "green" odor which mainly change cluster depending on the clustering method. Results from the others methods were discussed on S1 File.

### Chemical structures and functions of odorants

**Fig 6:**
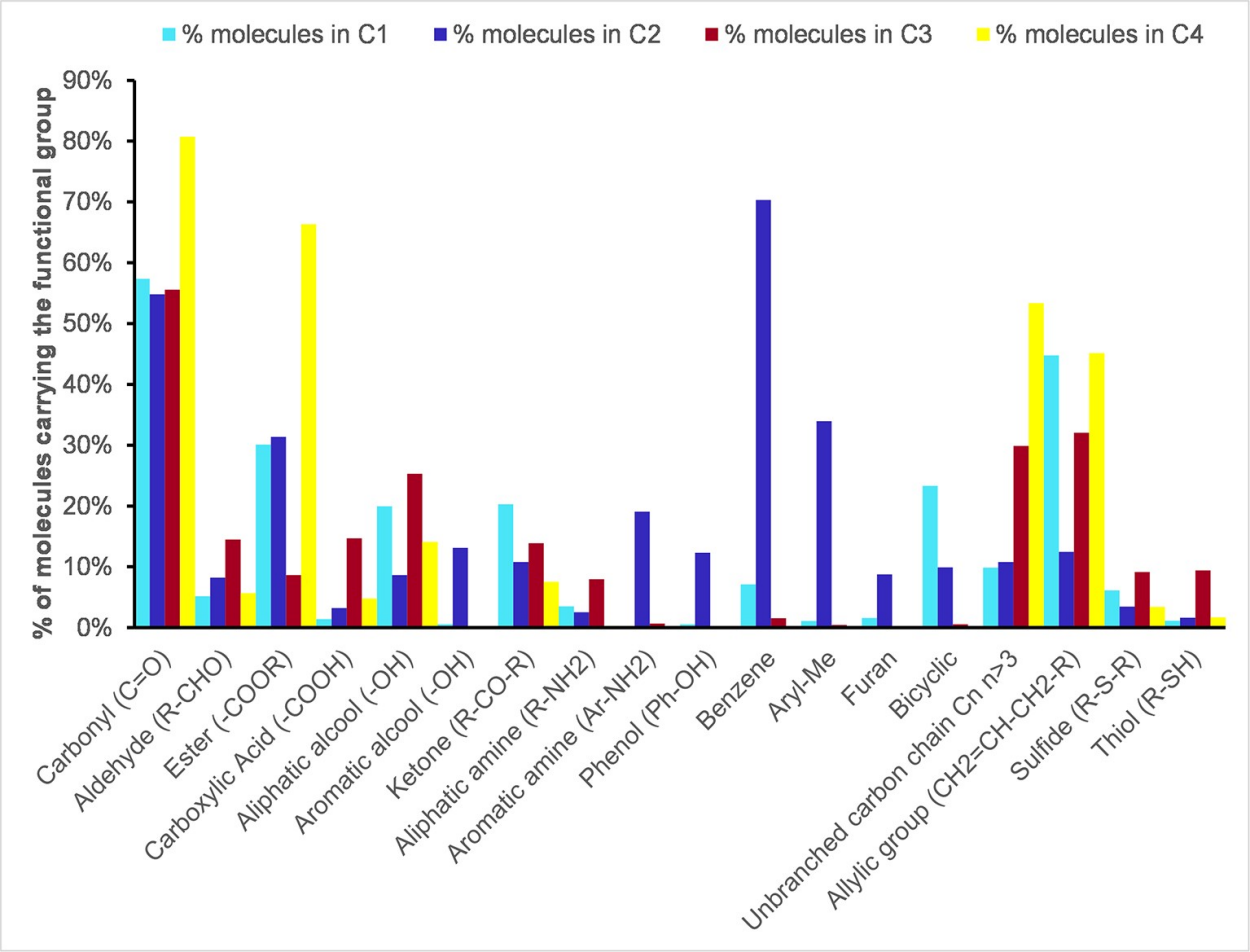
Histogram of the distribution of the chemical functional groups according the clusters. Only the structures present in at least 5% of the molecules of one of the 4 clusters C1, C2, C3 and C4 are represented: C1 in light blue; C2 in dark blue; C3 in dark red; C4 in yellow.

**Fig 7:**
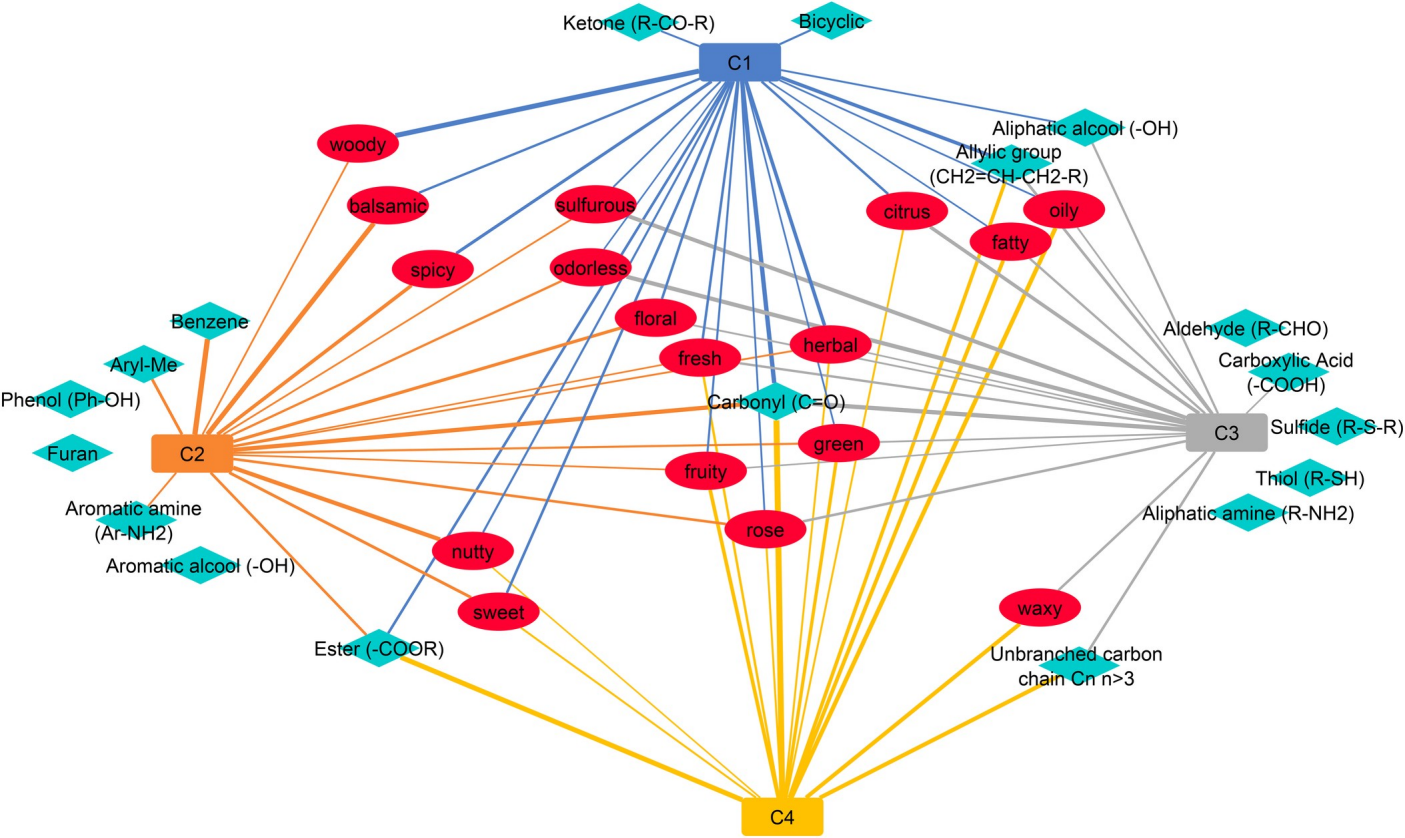
Network representation of the links between odor notes (red ellipse) and chemical functional groups (blue diamond). The nature of the line varies as a function of the relative frequency of occurrences. The thicker the line, the higher is the number of occurrences of an odor note or a chemical functional group within the cluster to which it is linked. The edges are invisibly for the relative frequency of occurrences less than 0.1. The blue, orange, grey and yellow rectangles correspond respectively to clusters 1, 2, 3 and 4. The blue lines correspond to the associations between the cluster 1 and the odor notes or the cluster 1 and the chemical functional groups. The orange lines correspond to the associations between the cluster 2 and the odor notes or the cluster 2 and the chemical functional groups. The grey lines correspond to the associations between the cluster 3 and the odor notes or the cluster 3 and the chemical functional groups. The yellow lines correspond to the associations between the cluster 4 and the odor notes or the cluster 4 and the chemical functional groups.

Among the 62 chemical structures and functions of different nature shared by the smell compounds of the dataset, we selected eighteen chemical functional groups present in at least 5% of the molecules of one of the 4 clusters (S3 Table). By focusing on these eighteen chemical structures and functions, we explored their frequency depending on the clusters to which they belong. As shown in Figs 6 and 7, carbonyl compounds were predominantly present in all clusters, that was the majority of odorant molecules have carbonyl groups, and the cluster 4 owned the higher percentage (80%), mainly as ester functions. Aldehydes and alcohols were mainly in cluster 3, as well as carboxylic acids, aliphatic amines, thiols and sulfides.

Molecules having an allylic group were especially frequent in clusters 1 and 4 (45%), and to a lesser extent in cluster 3. Moreover, the cluster C1 was especially rich in bicyclic structures. The cluster C4 was characterized by molecules with long carbon chains without ramifications (60%). Conversely, the cluster C2 was lacking in allyl groups, but was remarkably rich in unsaturated rings (phenols, aryl-methyl groups, aromatic amines and alcohols, furans) while molecules belonging to other clusters were deficient in such chemical groups.

## Discussion

Odor structure relationships in olfaction are key elements in understanding the olfactory system, an area in which there is still a great lack of knowledge [35–37].

With the aim to highlight the links between the molecular structure of smell compounds and their odor notes, we assessed four-dimensional reduction techniques applied to the molecular structures of 6038 smell compounds encoded by 1024- bit fingerprints. The spreading of smell compounds in a two-dimensional space was thus obtained for each technique. The coordinates were then used, independently, to perform a k-means and a AHC clustering, therefore providing the distribution of the smell compounds among several clusters. The visualization of the data in 2D spaces (Fig 3) showed the various areas defined by the clustering calculations, that allowed to evaluate the performance of the eight used approaches (reduction combined to clustering) to establish reliable links between molecular structures and odor notes (Figs 3–5). The less significant results were obtained using the t-SNE, as well concerning the blurred spatial arrangement of the elements in the 2D-space than the overlapping of clustering partitions obtained by k-means and AHC. The MDS and PCA calculations provided better but average results, except for PCA-AHC for which results were a slightly better. All the results and analyses put forward the precision of UMAP in aggregations of the elements according to the cluster areas that were reflected by the high degree of specificity of odor notes regarding the clusters. Indeed, as UMAP is based on the fact that manifold structure exists in the data, UMAP calculation is able to find these structures in the noise of a dataset which is suitable for data visualization. As the amount of data sampled increases, the amount of structure highlighted by noise lower [49]; therefore, the robustness of UMAP increases with the amount of data. Lastly, UMAP has the advantage of preserving the local and the global data structure, by keeping a runtime shorter than other dimension reduction techniques [60].

The characteristics of smell compounds across the UMAP clusters were examined on two points of view: the odor notes and the chemical functional groups. Analyzing the proportions of odor notes across the clusters focused on the 17 most frequent odor notes, including “odorless” quality. In parallel, 18 chemical functional groups were used to point out the main chemical features of the smell compounds. This dual approach revealed interesting specificities of the molecules according to the cluster to which they belong. The radar charts reported in Fig 4G and 4H and in S4 Fig bring out very distinct odor profiles. Few molecules of the combined clusters C2g1h shared the odor note “woody” and are characterized by allylic chains and carbonyl and ketone chemical functions. We noted that nearly 50 molecules carried both the odor notes “woody” and “spicy”; for example, copaene (woody; spicy), thujopsene (woody;spicy;dry), isocaryophyllene (woody; spicy), which are polycyclic molecules. The odor note “spicy” was rather frequent in C2g1h, and “balsamic” was the major odor note of C1g2h while the cyclic and aromatic moieties were a distinctiveness of the molecules of C1g2h. Interestingly, the bicyclic molecules were specific to some molecules of C1g2h and C2g1h, and quite absent from the clusters C3gh and C4gh. The odor notes “nutty” and “floral”, as well as “rose”, were also characteristic of molecules of C1g2h (S2 Table). Taking together the observations related to the clusters C1g2h and C2g1h, these suggested that two types of “spicy” molecules could be discriminated both by their perception and their structures: the "spicy-woody" and the "spicy-balsamic" molecules.

The cluster C3gh is peculiar in that “sulfurous” and citrus” molecules were mixed whereas “sulfurous” and “citrus” odors evoke opposing hedonic values unpleasant/pleasant [61]. C3gh is characterized by its composition on aldehydes, aliphatic alcohols and amines, carboxylic acids, and obviously organic sulfur molecules that share the sulfurous, sulfur and pungent odors. At the difference, there were very few esters. We can also note that odorless compounds that contribute to C3gh are amino acids, carboxylic acids and their salts. If excluding the effect of sulfur atom on the odor of “sulfurous” molecules, some structural features common to the carbon chains of “sulfurous” and “citrus” molecules could explain their grouping in C3gh. Further accurate examinations of the chemical structures will be needed to address this issue. The molecules that belonged to C4gh have “fruity”, “green”, “fatty” and “waxy” odor notes. As shown in a previous work [62] these odor notes were often used together in the descriptions of natural fruity odors of esters while long chains confer fatty and waxy odors. Indeed, about 50% of molecules of C4gh shared allylic or aliphatic chains, and ester function. Besides the odor “fruity” was frequently associated to “green” or “apple” in the odor descriptions, and less frequently to “fatty” or “waxy”. Obviously, no odor notes or chemical structure were specific to a cluster, which was not surprising, but it was still possible to associate certain chemical structures with certain odors (S4 Table). It could not be expected to adjust in only four groups the complexity of many thousands of odorants and several millions of perceptible odors [63]. Moreover, most molecules were described by 3 or 4 odor notes (Fig 2A), meaning that there exist “spicy-woody” and “spicy-balsamic”, “fruity-green” and “fruity-fatty” molecules, and numerous other cases [62], and that these odors can be discriminated by humans. Such associations of odors notes will be considered in a further work.

To conclude, the obtained results highlight some relationships between the structure of the molecule and odor. The UMAP dimensional reduction method associated to k-means and AHC clustering techniques allowed to obtain interesting results revealing links between molecular structures and odor qualities. Such association of k-means and AHC clustering with UMAP is the first performed on molecular fingerprints for a dataset related to odors. Therefore, the use of UMAP provides a promising way to improve the understanding of the structure-odor relationships by visualizing high quality embedding of large data sets that were previously unattainable [49]. Upcoming studies would be considered to refine the odor-structure relationships inside specific group by applying other clustering methods as Maximum Common Substructure Methods or Gaussian mixture model [64, 65]. In perspective, it would be interesting to integrate olfactory receptors on which odorant molecules interact to, in order to demonstrate structure-odor-receptor relationships. In addition, conducting this study using a 3-D dimensional reduction could provide complementary information on the structure-odor relationships as an extension of the present study.

## Supporting information

S1 Table Fingerprint, coordinates in 2D spaces and clusters. (XLSX)

S2 Table Odor notes and occurrences. (XLSX)

S3 Table Distribution of the chemical groups and functions by cluster. (DOCX)

S4 Table Table of chemical structures associated with odors. (DOCX)

S1 Fig “Elbow” curve. Representation of intra-cluster variability as a function of the number of clusters. The optimal number of clusters is around the bend of the curve.
(DOCX)

S2 Fig Progression of the penalty score according to the number of clusters. The minimum score is assigned to the optimal number of clusters.
(DOCX)

S3 Fig Dendrograms of the AHC of molecules for each dimension reduction technique. (DOCX)

S4 Fig Radar charts of the distribution of the %ON values obtained for the 17 most frequent odor notes across clusters of the UMAP-kmeans and UMAP-AHC techniques. A: Comparison between C1g and C2h. B: Comparison between C2g and C1h. C: Comparison between C3g and C3h. D: Comparison between C1g and C2h.
(DOCX)

S1 File (DOCX)
