# Supplementary material for: Smell compounds classification using UMAP to increase knowledge of odors and molecular structures linkages
Source: PLoS One. 2021 May 28;16(5):e0252486. doi: 10.1371/journal.pone.0252486 (PMC8162648; doi:10.1371/journal.pone.0252486)
Supplement: S3 Table — (DOCX) [file pone.0252486.s003.docx]

**S3 Table. Distribution of the chemical groups and functions by cluster.**

| Chemical function | % molecules in C1 | % molecules in C2 | % molecules in C3 | % molecules in C4 |
| --- | --- | --- | --- | --- |
| Carbonyl (C=O) | 57,37% | 54,80% | 55,56% | 80,70% |
| Aldehyde (R-CHO) | 5,18% | 8,21% | 14,49% | 5,67% |
| Ester (-COOR) | 30,09% | 31,38% | 8,63% | 66,33% |
| Carboxylic Acid (-COOH) | 1,40% | 3,22% | 14,71% | 4,79% |
| Aliphatic alcool (-OH) | 19,98% | 8,65% | 25,30% | 14,10% |
| Aromatic alcool (-OH) | 0,55% | 13,13% | 0,08% | 0,00% |
| Ketone (R-CO-R) | 20,28% | 10,80% | 13,89% | 7,56% |
| Aliphatic amine (R-NH2) | 3,53% | 2,53% | 7,96% | 0,27% |
| Aromatic amine (Ar-NH2) | 0,12% | 19,07% | 0,68% | 0,00% |
| Phenol (Ph-OH) | 0,55% | 12,31% | 0,08% | 0,00% |
| Benzene | 7,13% | 70,33% | 1,58% | 0,00% |
| Aryl-Me | 1,10% | 33,96% | 0,45% | 0,00% |
| Furan | 1,58% | 8,78% | 0,15% | 0,00% |
| Bicyclic | 23,33% | 9,91% | 0,53% | 0,00% |
| Unbranched carbon chain Cn n>3 | 9,87% | 10,80% | 29,88% | 53,37% |
| Allylic group (CH2=CH-CH2-R) | 44,76% | 12,44% | 32,06% | 45,14% |
| Sulfide (R-S-R) | 6,15% | 3,47% | 9,16% | 3,44% |
| Thiol (R-SH) | 1,16% | 1,64% | 9,38% | 1,69% |
