# Supplementary material for: Smell compounds classification using UMAP to increase knowledge of odors and molecular structures linkages
Source: PLoS One. 2021 May 28;16(5):e0252486. doi: 10.1371/journal.pone.0252486 (PMC8162648; doi:10.1371/journal.pone.0252486)
Supplement: S4 Table — (DOCX) [file pone.0252486.s004.docx]

**S4 Table. Table of chemical structures associated with odors.**

| Chemical group | Odor |
| --- | --- |
| Long chain | Fatty |
| Long chain | Waxy |
| Amino acids, carboxylic acids | Odorless |
| Ester | Fruity |
| Sulfur | Sulfurous |
| Sulfur | Pungent |
| Polycyclic | Woody, spicy |
| Allylic chain, carbonyl, ketone | Woody |
