# Supplementary material for: Smell compounds classification using UMAP to increase knowledge of odors and molecular structures linkages
Source: PLoS One. 2021 May 28;16(5):e0252486. doi: 10.1371/journal.pone.0252486 (PMC8162648; doi:10.1371/journal.pone.0252486)
Supplement: S1 Fig — Representation of intra-cluster variability as a function of the number of clusters. The optimal number of clusters is around the bend of the curve. (DOCX) [file pone.0252486.s005.docx]

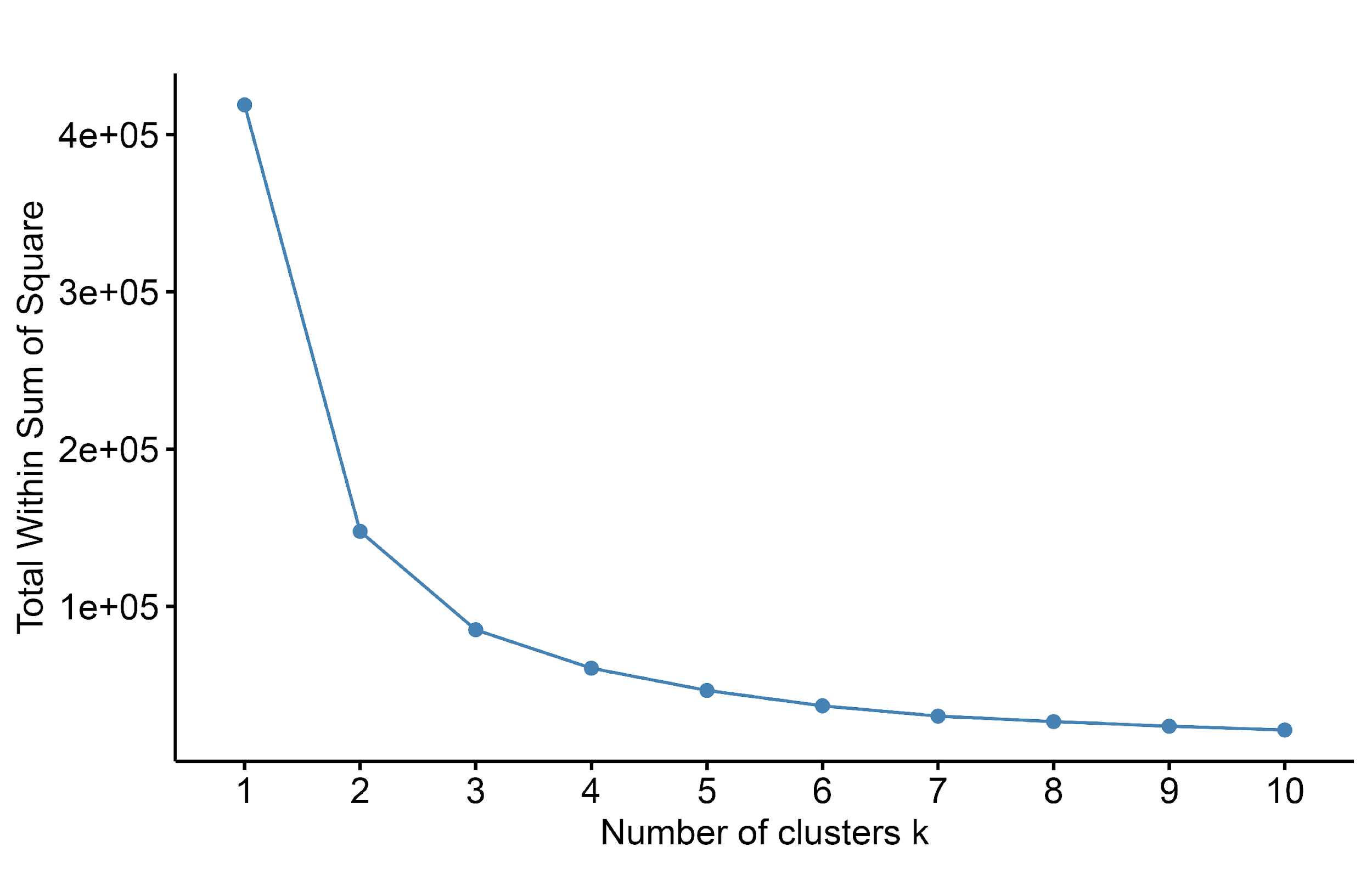

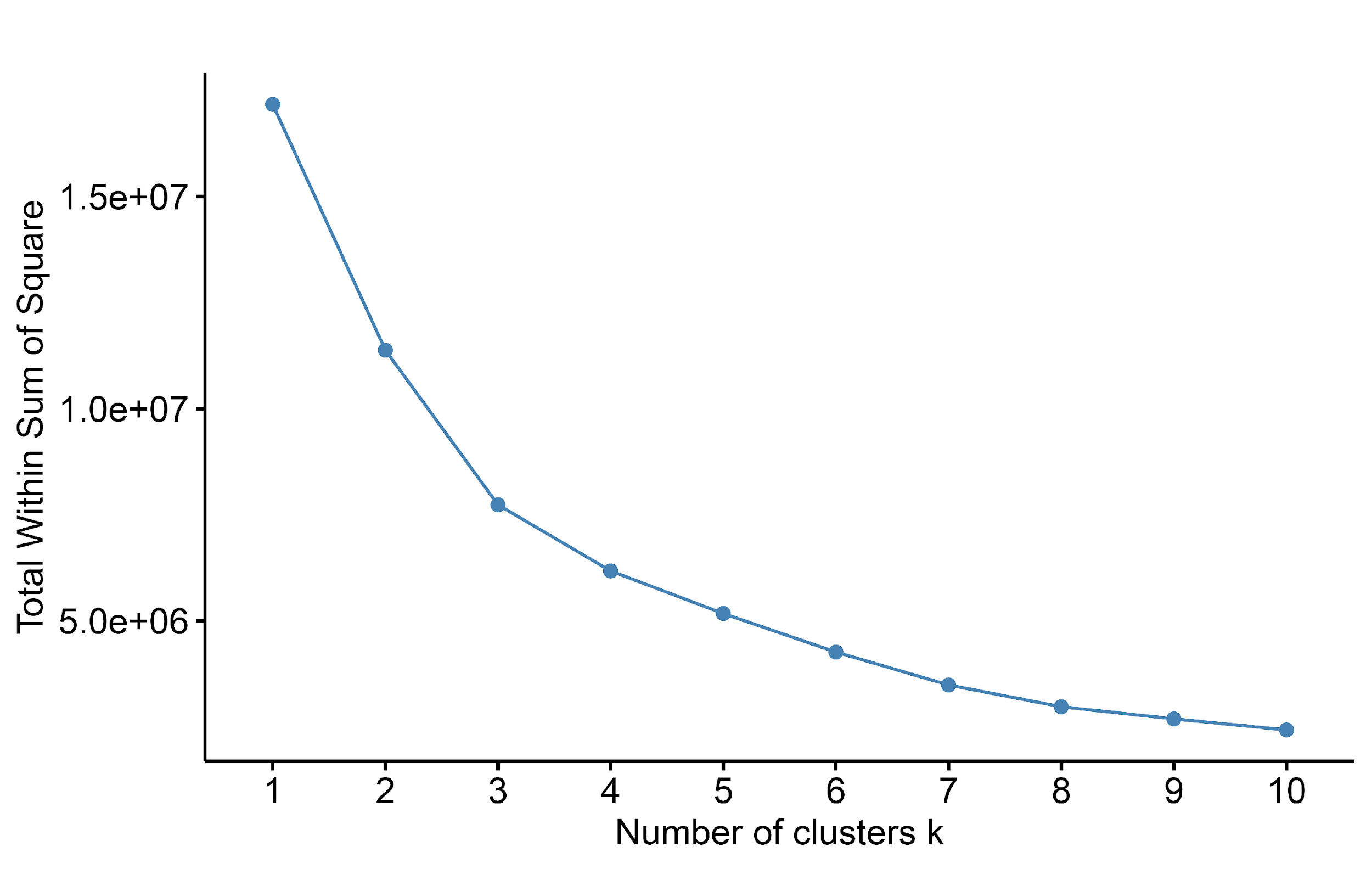

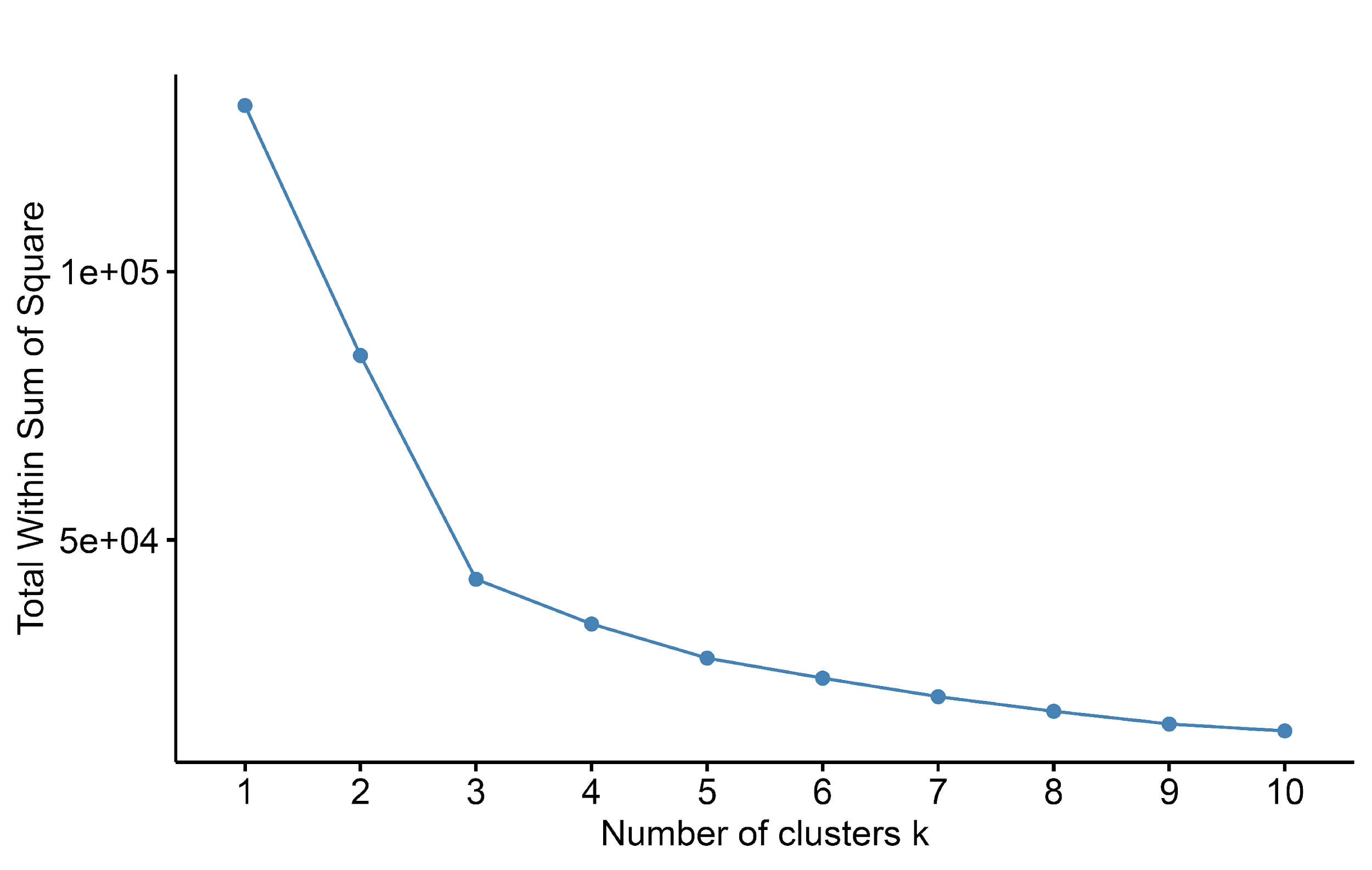


PCA


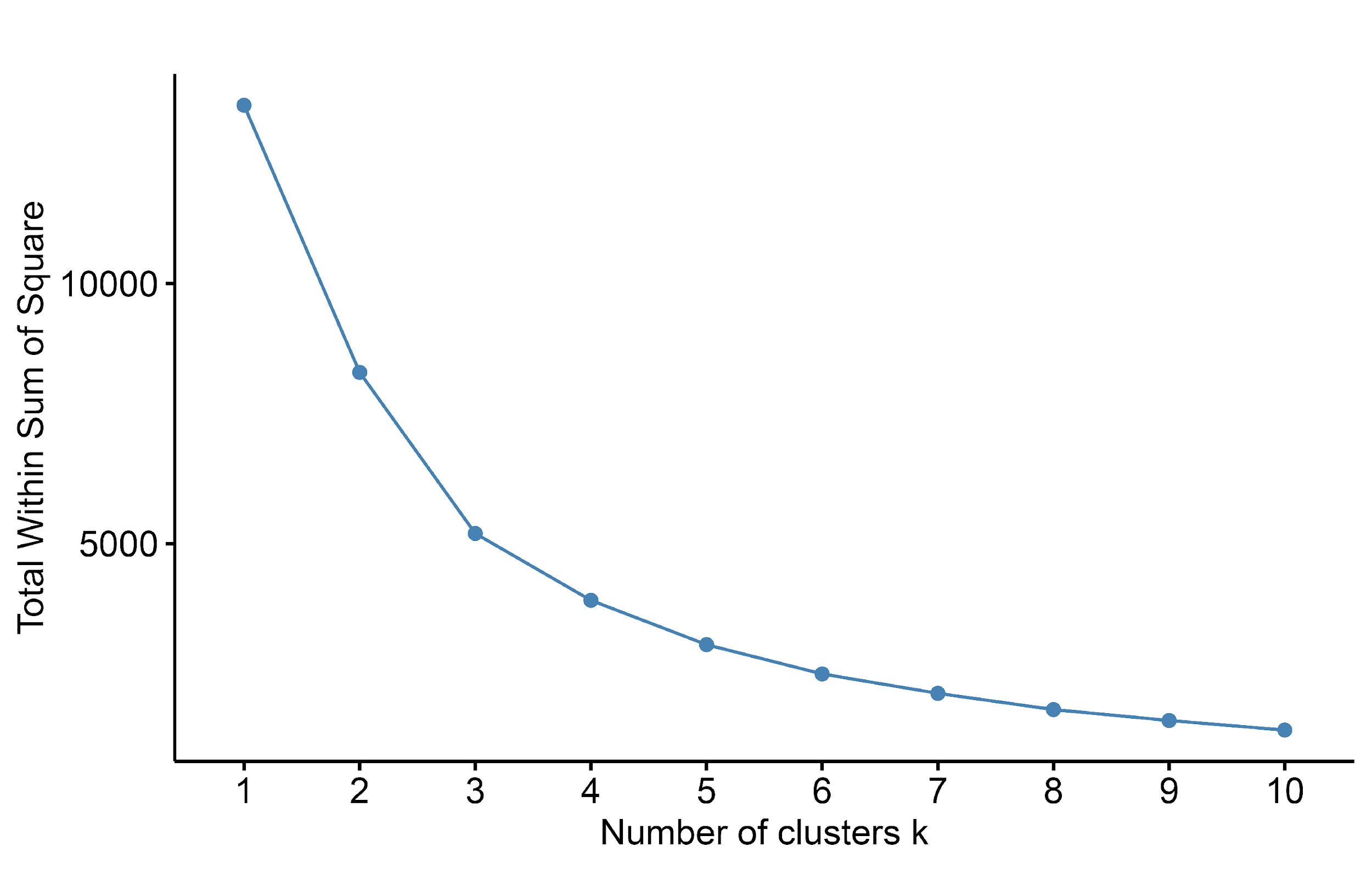


MDS

t-SNE

UMAP

**S1 Fig. “Elbow” curve.**

Representation of intra-cluster variability as a function of the number of clusters. The optimal number of clusters is around the bend of the curve.
