# Supplementary material for: Smell compounds classification using UMAP to increase knowledge of odors and molecular structures linkages
Source: PLoS One. 2021 May 28;16(5):e0252486. doi: 10.1371/journal.pone.0252486 (PMC8162648; doi:10.1371/journal.pone.0252486)
Supplement: S2 Fig — The minimum score is assigned to the optimal number of clusters. (DOCX) [file pone.0252486.s006.docx]

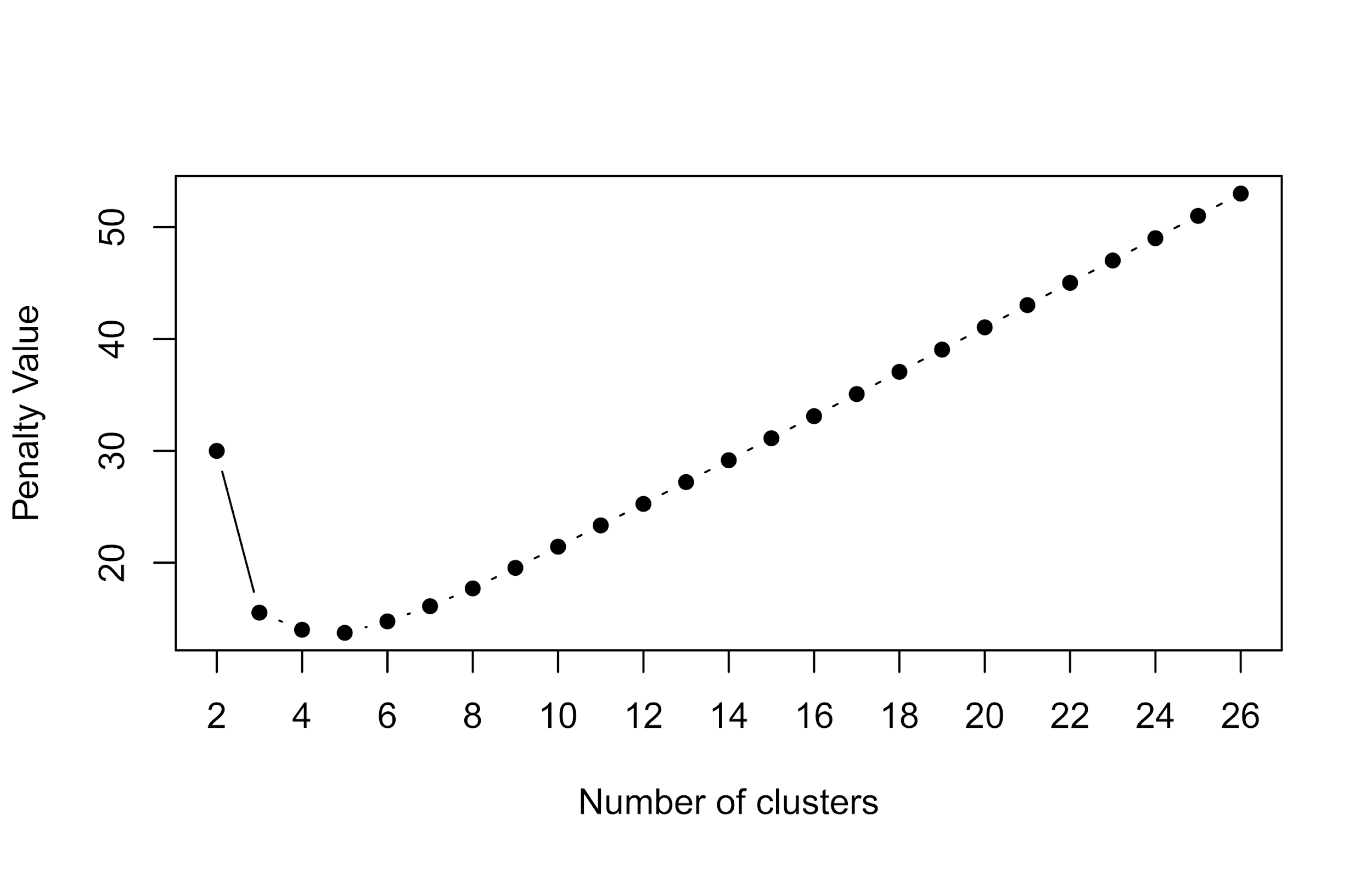

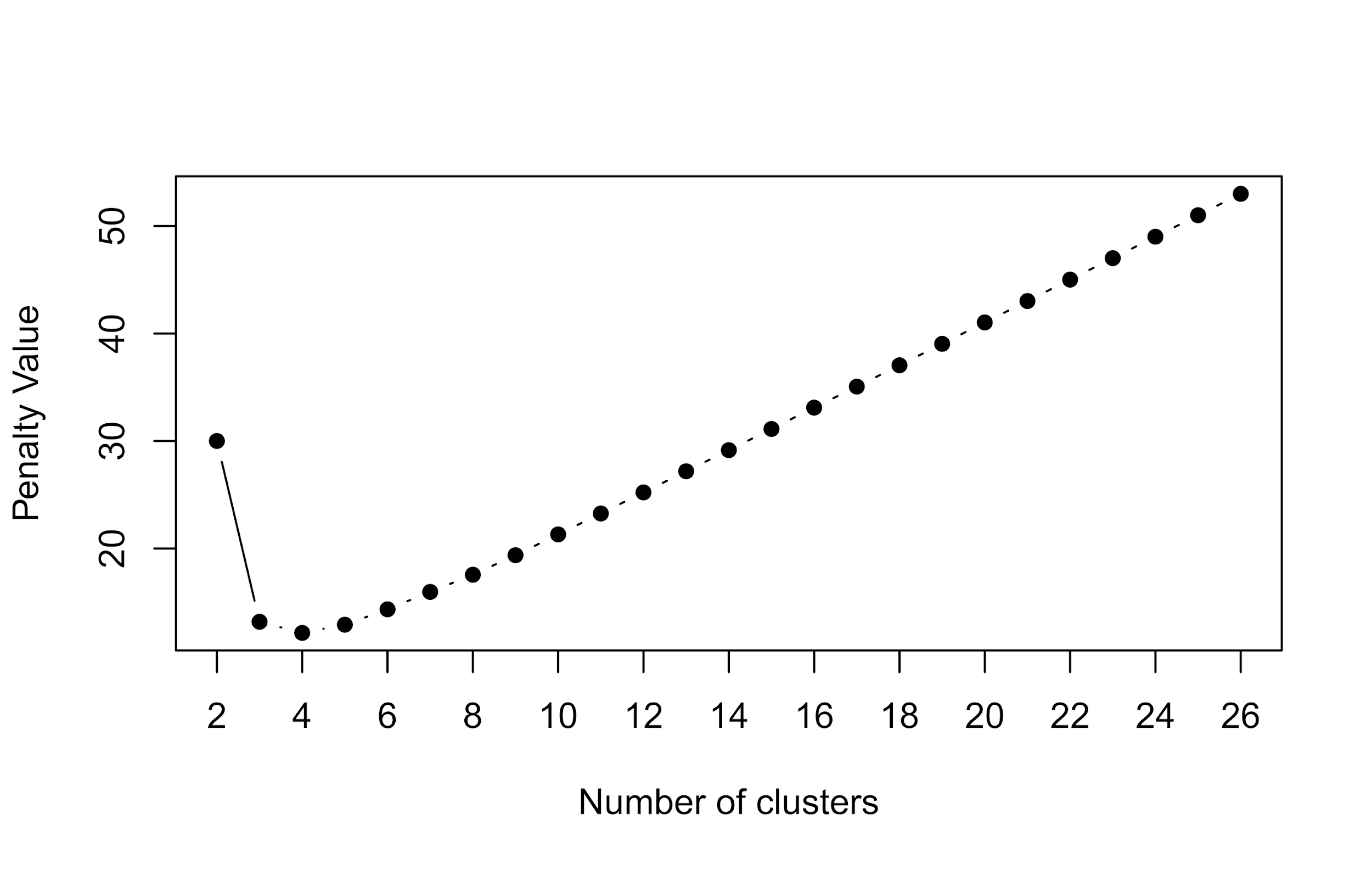

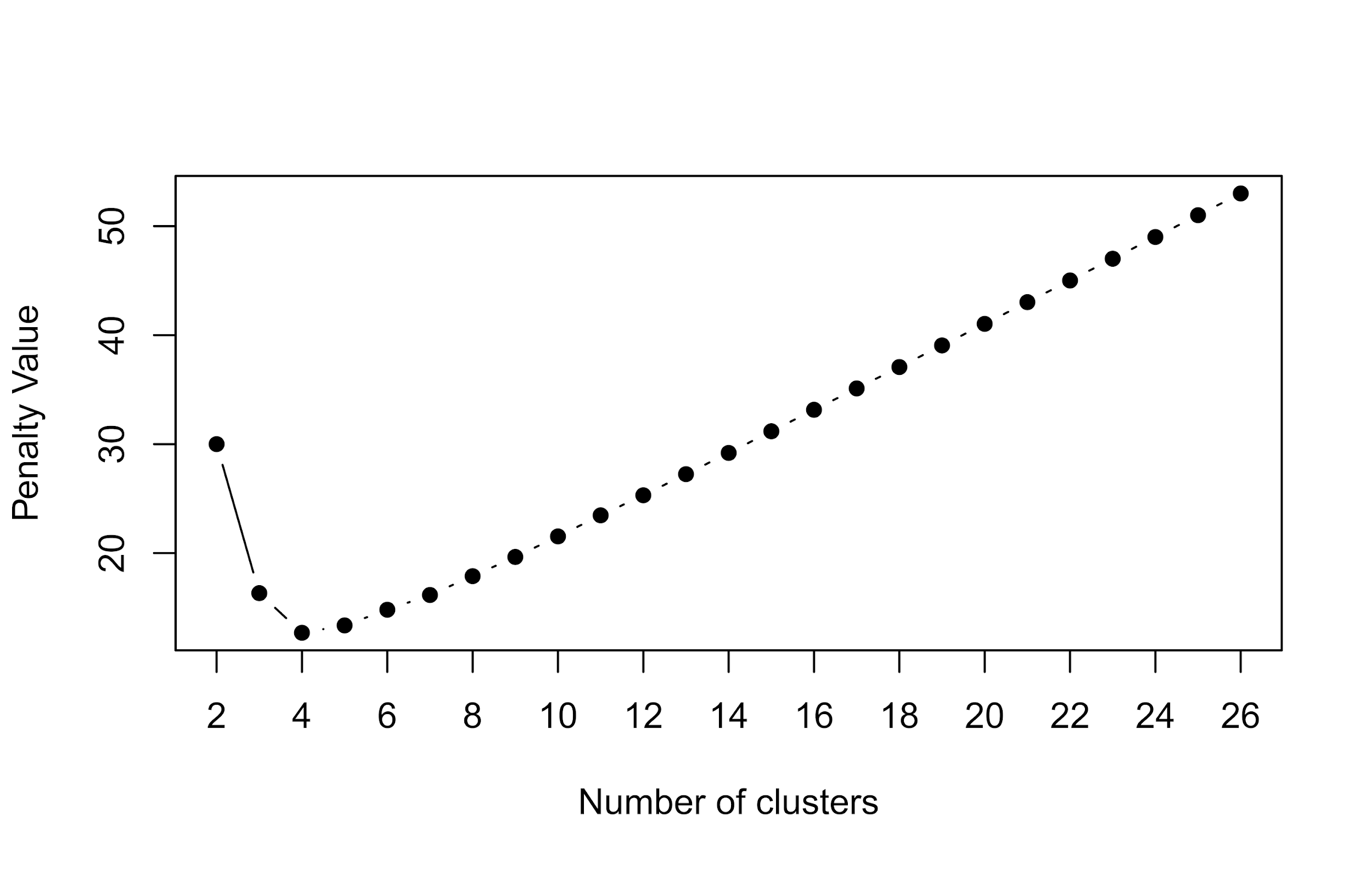

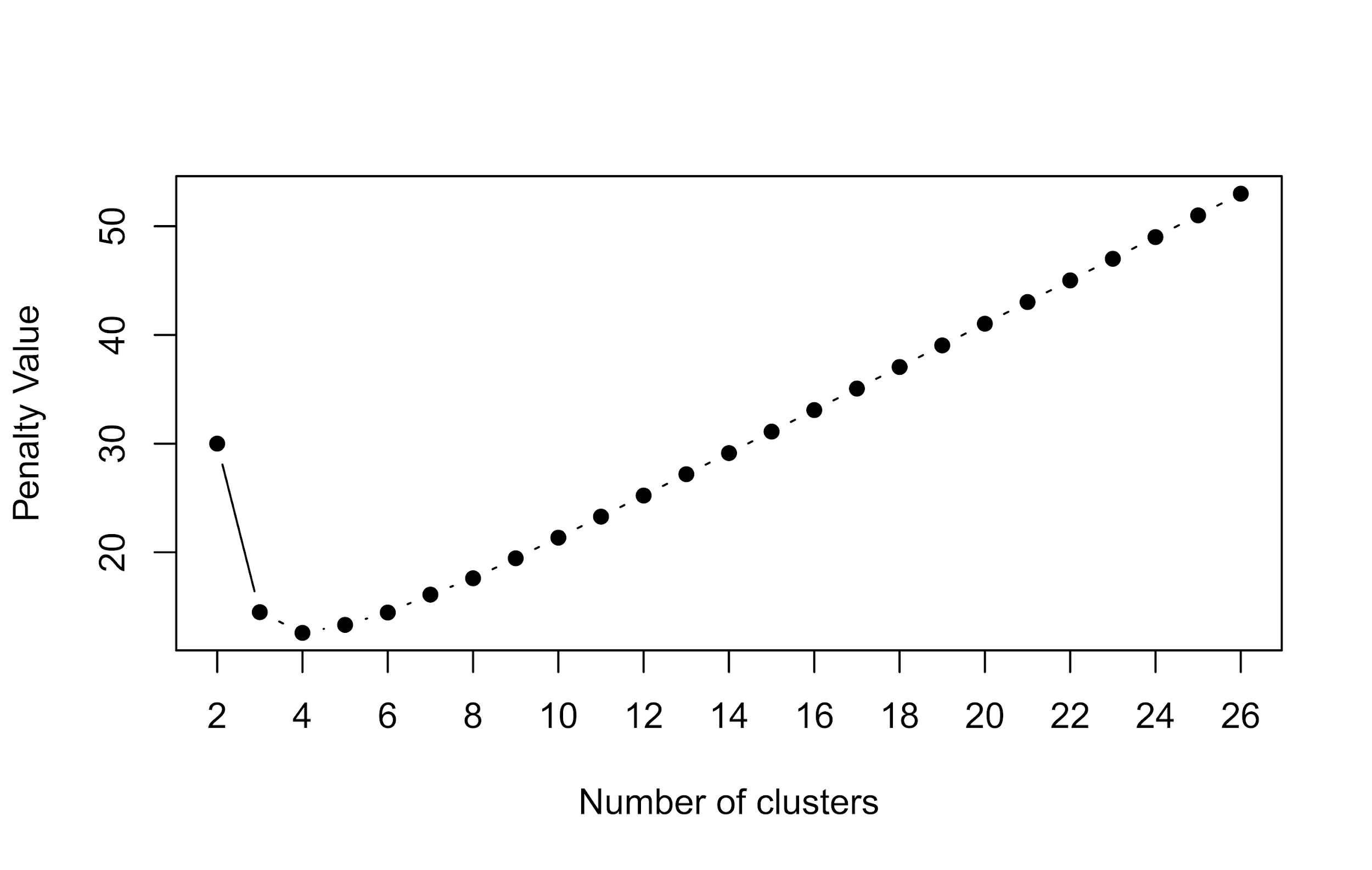


PCA

t-SNE

UMAP

MDS

**S2 Fig. Progression of the penalty score according to the number of clusters.**

The minimum score is assigned to the optimal number of clusters.
