# Supplementary material for: Smell compounds classification using UMAP to increase knowledge of odors and molecular structures linkages
Source: PLoS One. 2021 May 28;16(5):e0252486. doi: 10.1371/journal.pone.0252486 (PMC8162648; doi:10.1371/journal.pone.0252486)
Supplement: S3 Fig — (DOCX) [file pone.0252486.s007.docx]

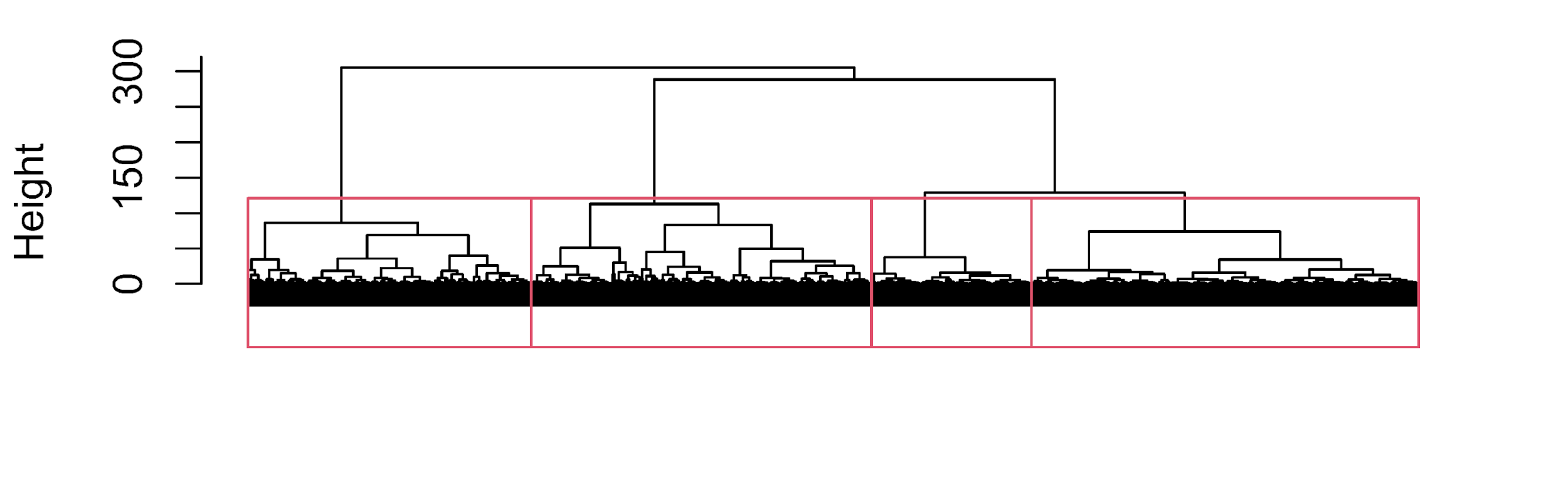


PCA

MDS

t-SNE

UMAP


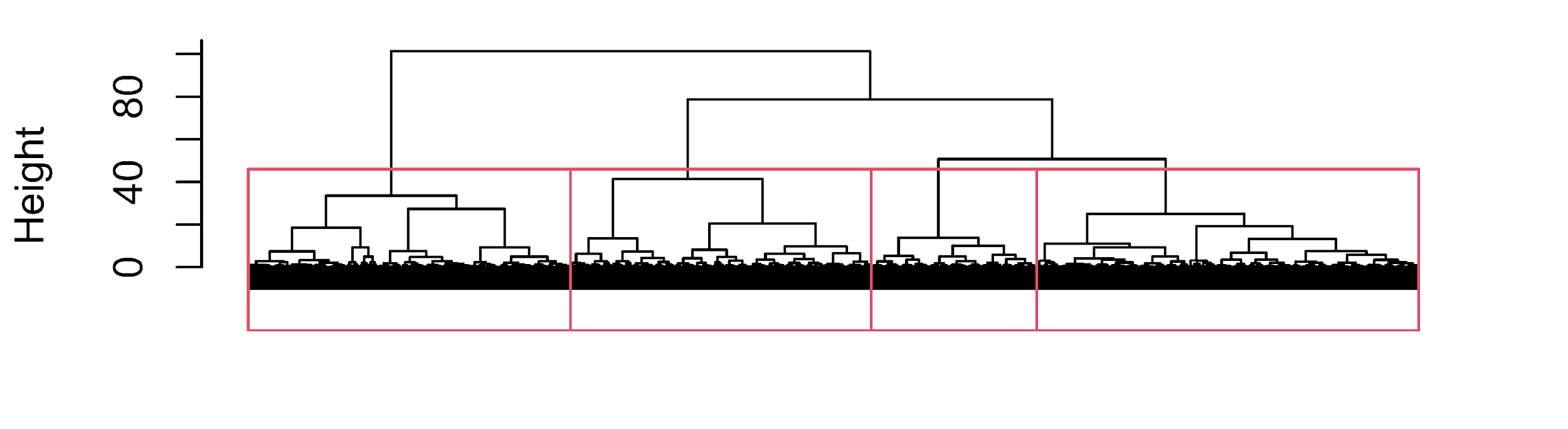

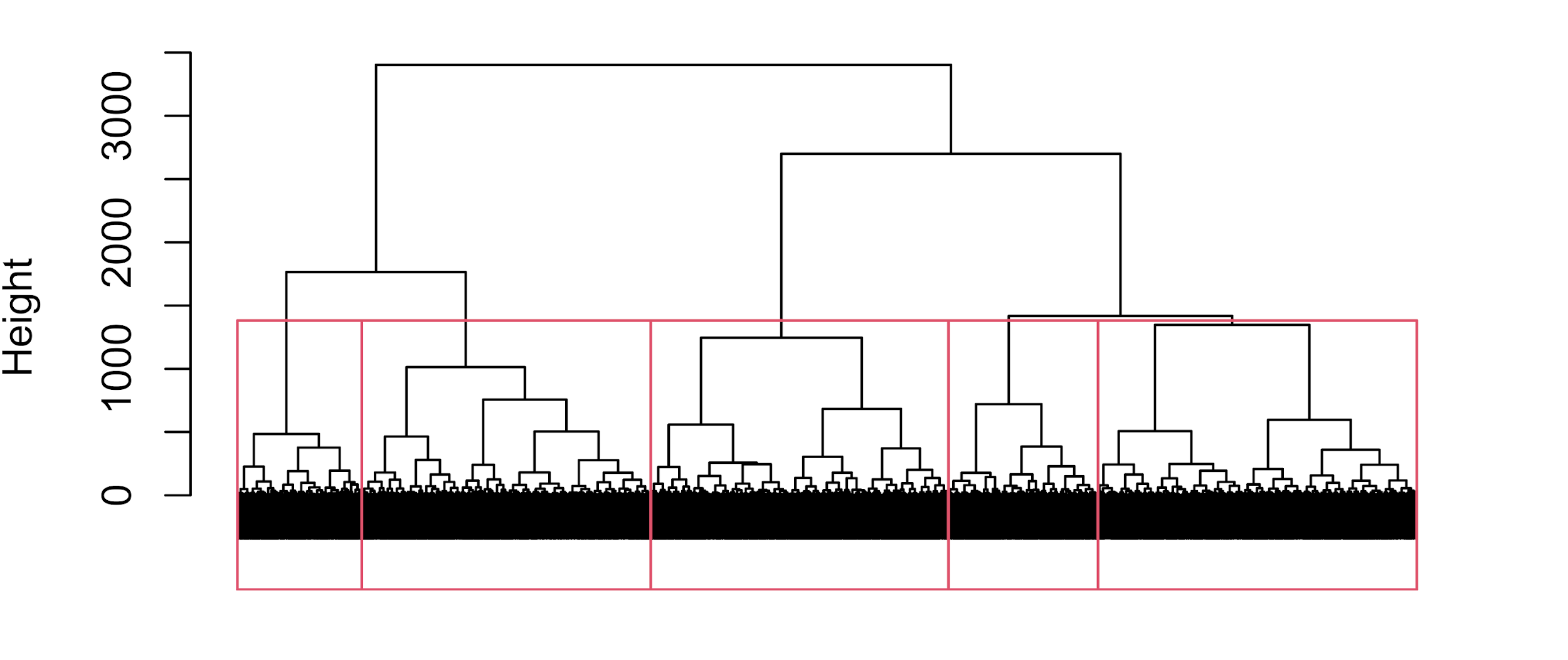

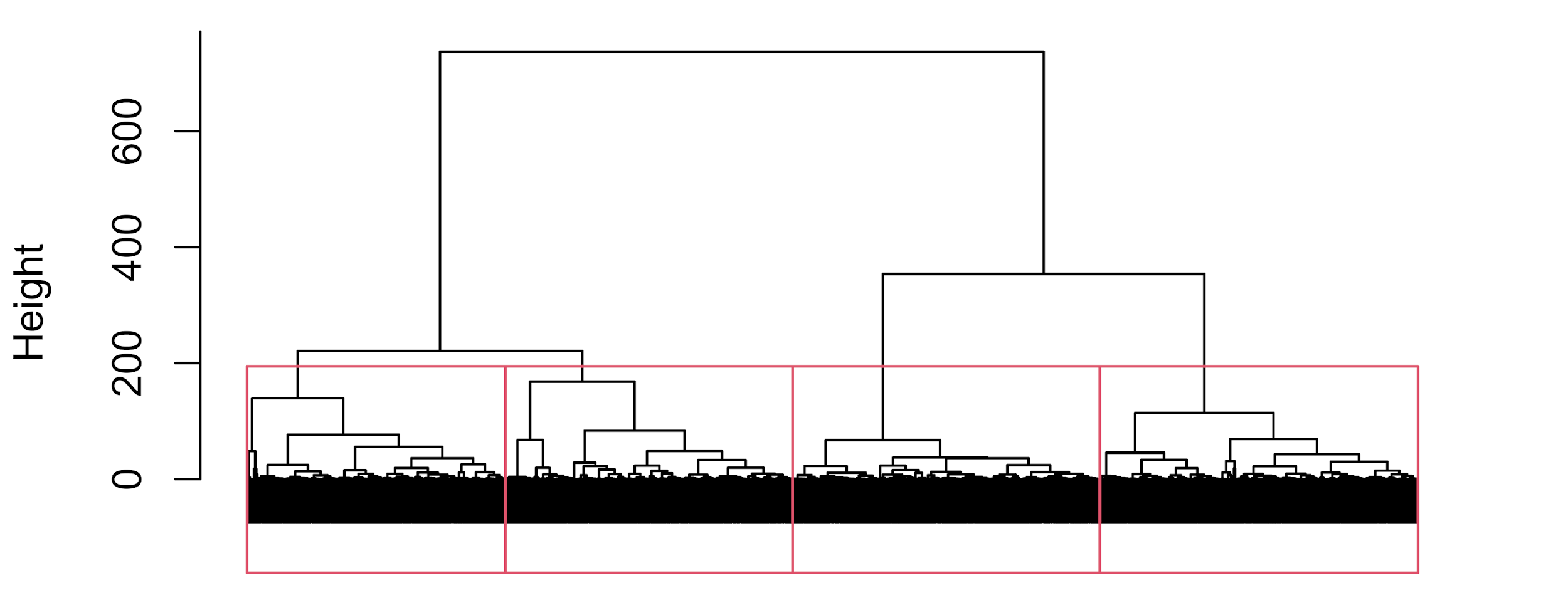


Cluster 1

Cluster 1

Cluster 1

Cluster 1

Cluster 2

Cluster 2

Cluster 2

Cluster 2

Cluster 3

Cluster 3

Cluster 3

Cluster 3

Cluster 4

Cluster 4

Cluster 4

Cluster 4

Cluster 5

**S3 Fig. Dendrograms of the AHC of molecules for each dimension reduction technique.**
