# Supplementary material for: Smell compounds classification using UMAP to increase knowledge of odors and molecular structures linkages
Source: PLoS One. 2021 May 28;16(5):e0252486. doi: 10.1371/journal.pone.0252486 (PMC8162648; doi:10.1371/journal.pone.0252486)
Supplement: S4 Fig — A: Comparison between C1g and C2h. B: Comparison between C2g and C1h. C: Comparison between C3g and C3h. D: Comparison between C1g and C2h. (DOCX) [file pone.0252486.s008.docx]

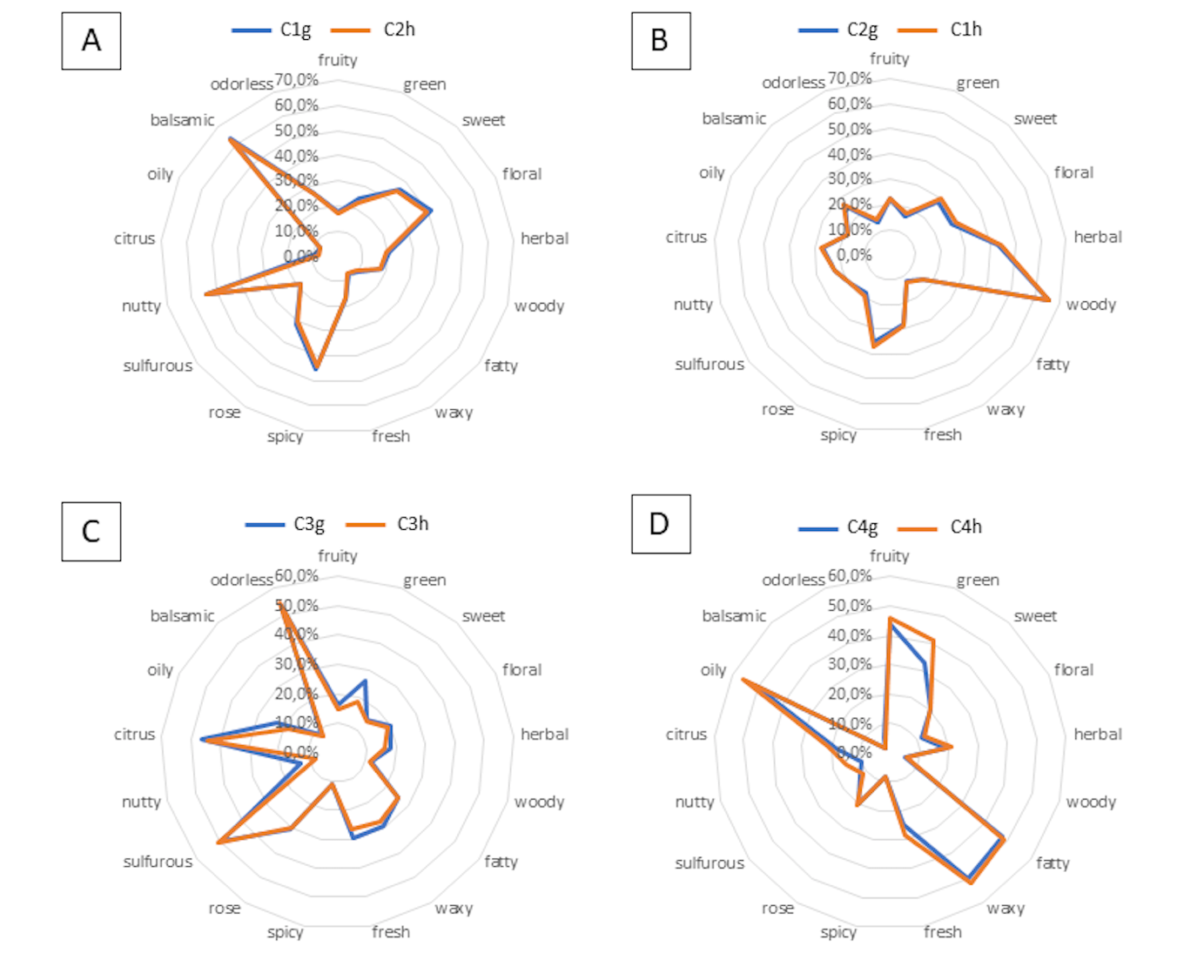


**S4 Fig. Radar charts of the distribution of the %ON values obtained for the 17 most frequent odor notes across clusters of the UMAP-kmeans and UMAP-AHC techniques.**

A: Comparison between C1g and C2h. B: Comparison between C2g and C1h. C: Comparison between C3g and C3h. D: Comparison between C4g and C4h.
