# Supplementary material for: Smell compounds classification using UMAP to increase knowledge of odors and molecular structures linkages
Source: PLoS One. 2021 May 28;16(5):e0252486. doi: 10.1371/journal.pone.0252486 (PMC8162648; doi:10.1371/journal.pone.0252486)
Supplement: S1 File — (DOCX) [file pone.0252486.s009.docx]

**S1 File**

**Description of results obtained with PCA, MDS, t-SNE coupled with K-means and HCA on the odors distribution.**

The cluster C1a (PCA k-means) brings together more than 60% of “fatty” and “waxy” odor notes, while “woody” is especially frequent in C1b (PCA AHC). No cluster PCA AHC carried more than 50% of “fatty” and “waxy” odors. Both the clusters C2a (PCA k-means) and C2b (PCA AHC) were enriched in “balsamic”, “floral”, “spicy” and “nutty” odor notes although for “balsamic” this effect was less pronounced with C2a (53% of its occurrences) than with C2b (%ON reaches 60%). “Odorless” and “sulfurous” odor notes were mainly in C3a and C3b, but “citrus” and “green” were two fold more present in C3b than in C3a (“citrus” note was more frequent in C1a). “Balsamic”, “floral”, “spicy” and “nutty” odor notes characterized the clusters C1c (MDS k-means) and C2d (MDS AHC). The “fatty”, “waxy”, “oily” and “rose” were distributed according to similar profiles in both C3c and C4d. “Odorless” and “sulfurous” odor notes were mainly in C2c and C3d, and “citrus” notes were also in these clusters. “Woody” notes were in C4c and C1d, but while 40% of “nutty” notes were in C4c, only 13% were in C1d and mainly in C2d (36% of occurrences). Clustering calculations were provided for five clusters using t-SNE coordinates in 2D-space. The notes “woody”, “spicy” and “nutty” are mainly in C1e(t-SNE k-means) and C1f(t-SNE AHC). Nevertheless, while these three notes were in equivalent proportions in C1e, “spicy” was mainly distributed between C1f and C2f, and to a lesser degree between C3f and C4f. “Balsamic” and “floral” notes were essentially in C3e and C5f. The cluster C3e and C5f encompassed more than 30% of “rose” notes, but 33% of “spicy” occurrences were in C3e as compared to 13% in C5f. The “odorless” were mainly in C2e and C3f, however 44% of occurrences of “sulfurous” are in C3e, only 27% were in C3f. Conversely, “fruity” and “oily” notes that were rare in C3e, gathered near 40% of their occurrences in C5f. “Fatty” and “waxy” notes were present between C4e, C5e and C4f, but also in C3f along “odorless” notes. The cluster C3e was characterized by “fatty”, “citrus and “green” notes, while “fruity”, “waxy”, “rose” and “oily” were mostly abundant in C5e.

**Results of molecules in the intersection between 2 clusters obtained with PCA, MDS, t-SNE coupled with K-means and HCA.**

Several molecules belonging to the cluster C2b (PCA AHC) were attributed to C1a (PCA k-means). Thus, 46 molecules belong to C1a (PCA k-means)∩C2b(PCA AHC) (Fig 3a and 3b). The main notes presented in C1a are “fatty”, “waxy” “oily”, whereas the cluster C2b was dominated by “balsamic”, “spicy”, “floral” and “sweet” odors. Interestingly, the 46 molecules present the odor notes “floral”, “rose”, “sweet”, “balsamic” and “waxy”. Their position to the C2b region of the PCA map was coherent with their “balsamic- floral” character, while the note “waxy” explained their association to C1a. A similar finding was identified for clusters C4c(MDS k-means)∩C2d(MDS AHC). The main notes of C4c (MDS k-means) were “woody”, “spicy” and “nutty”, while “fatty”, “waxy” “oily” characterized the molecules belonging to C2d (MDS AHC). 344 molecules belonging to C4c were also associated to C2d, and shared the notes “woody”, “nutty”, “spicy” typical of the cluster C4c. Regarding clusters obtained from t-SNE coordinates, some were especially intricate. As an example, we focused on the inter-clusters C5e(t-SNE k-means)∩C3f(t-SNE AHC) and C2e(k-means) ∩C4f(AHC). The group C5e(t-SNE k-means)∩C3f(t-SNE AHC) encompassed 896 molecules. The molecules of C5e were characterized by “fruity”, “rose”, “waxy” and “oily” odors, while “odorless” was especially frequent in the cluster C3f. The molecules that “shift” between the two clusters shared predominantly the odor notes “fruity”, “green”, “waxy”, “fatty”, “oily” and “floral”, and can be regarded as non-odorless molecules of C3e. C2e(k-means) ∩C4f(AHC) was a smallest group that comprises 475 elements. The molecules of C2e shared “sulfurous” and “odorless” notes, and molecules of C4f had “fatty”, “waxy”, and also “green”, “oily” and “citrus”. The intersection of C2e C4f was mainly “sulfurous” and “green” (representing 21% of molecules of the group), but there were only a few “odorless” and no citrus molecules.
